# Landscape and impact of mind-body, cognitive-behavioral, and physical activity interventions in adolescent and adult brain tumor patients: A systematic review

**DOI:** 10.1093/noajnl/vdae134

**Published:** 2024-08-22

**Authors:** Alex R Wollet, James L Rogers, Sefanit Berhanu, Ciara Locke, Madhura Managoli, Emily Wu, I Diane Cooper, Terri S Armstrong, Amanda L King

**Affiliations:** Neuro-Oncology Branch, Center for Cancer Research, National Cancer Institute, National Institutes of Health, Bethesda, Maryland, USA; Neuro-Oncology Branch, Center for Cancer Research, National Cancer Institute, National Institutes of Health, Bethesda, Maryland, USA; Neuro-Oncology Branch, Center for Cancer Research, National Cancer Institute, National Institutes of Health, Bethesda, Maryland, USA; Neuro-Oncology Branch, Center for Cancer Research, National Cancer Institute, National Institutes of Health, Bethesda, Maryland, USA; Neuro-Oncology Branch, Center for Cancer Research, National Cancer Institute, National Institutes of Health, Bethesda, Maryland, USA; Neuro-Oncology Branch, Center for Cancer Research, National Cancer Institute, National Institutes of Health, Bethesda, Maryland, USA; Office of Research Services, National Institutes of Health, Bethesda, Maryland, USA; Neuro-Oncology Branch, Center for Cancer Research, National Cancer Institute, National Institutes of Health, Bethesda, Maryland, USA; Neuro-Oncology Branch, Center for Cancer Research, National Cancer Institute, National Institutes of Health, Bethesda, Maryland, USA

**Keywords:** cognitive-behavioral, mind-body, neuro-oncology, physical activity, quality of life, symptoms

## Abstract

**Background:**

The use of mind-body, cognitive-behavioral, and physical activity interventions have shown efficacy for improving symptom burden and functional limitations in other cancers; however, these strategies have not been widely implemented within neuro-oncology. This systematic review describes the current landscape and the impact of these interventions on adolescent and adult patients with brain tumors, which may guide the development of future interventions.

**Methods:**

A systematic search of PubMed, Embase, and Web of Science was performed using preferred reporting items for systematic reviews and meta-analyses (PRISMA) guidelines with predefined eligibility criteria. Twenty-nine studies met the inclusion criteria and were selected for review.

**Results:**

There was promising evidence for the feasibility and efficacy of mind-body and physical activity interventions for improving mood and quality of life, as well as enhanced physical functioning following aerobic and strength-based interventions. Results were mixed for cognitive-behavioral interventions, likely due to underpowered analyses. Interventions tested in pediatric patients also showed improvements in fatigue, mood, and quality of life, though these individuals represented a small proportion of the pooled sample.

**Conclusions:**

Findings suggest that mind-body and physical activity interventions can improve both physical and psychological health for patients with brain tumors, though additional well-designed clinical trials are needed to better establish efficacy.

**Key Points**

- Evidence was promising for feasibility and efficacy of mind-body and physical activity interventions for improving mood and quality of life, as well as enhanced physical functioning following aerobic and strength-based interventions.
- Interventions for adolescent patients also showed improvements in fatigue, mood, and quality of life, though these individuals represented a small proportion of the pooled sample.
- Mind-body and physical activity interventions can improve both physical and psychological health for patients with brain tumors, though additional well-designed clinical trials are needed to better establish efficacy.

**Importance of the Study**

This review highlights the feasibility and efficacy of mind-body, cognitive-behavioral, and physical activity interventions for adolescent and adult patients with brain tumors, with the majority of included studies showing improvement in physical and/or psychological outcomes. In a highly symptomatic patient population with an often dismal prognosis, it is of the utmost importance to prioritize maximizing their quality of life and promoting healthy behaviors and strategies so that they can better self-manage symptoms and optimize their health.

Adolescent and adult patients with primary brain tumors (PBT) often encounter a challenging disease course, plagued by high symptom burden and functional interference that frequently persists from the time of initial diagnosis into the survivorship period.^1,2^ Symptoms may result from the tumor itself and/or tumor-directed treatments and often preclude patients from working, walking, performing usual activities, and enjoying life.^3^ It has been increasingly noted that PBT patients seek to not only live longer but also live higher quality lives.^4^ However, some neuro-oncology providers struggle to provide adequate care to PBT patients with particularly severe symptoms or functional limitations, resulting in fewer treatment and clinical trial options for these individuals.^5^ Therefore, PBT patients would benefit from interventions that seek to attenuate the debilitating physical, cognitive, and emotional symptoms that compromise their quality of life.^6,7^ Adolescents in particular have been historically overlooked in cancer research and may have unique needs^8,9^ and preferred interventional strategies for symptom management, which have yet to be explored in brain tumor patients.

Within neuro-oncology practice, few efficacious pharmacological-based symptom management interventions have been developed. Given bleak prognoses and severe symptoms, PBT patients have been documented to turn to complementary and alternative medicine treatments, but these approaches are often attempted without counsel or supervision by a healthcare professional and are not always based on robust evidence.^10^ Mind-body interventions are some of the most commonly used psychological approaches and focus on the relationships between the brain, mind, body, and behavior, and their impact on overall health,^11^ while cognitive-behavioral interventions aim to change thought patterns in order to promote positive behaviors.^12^ These therapeutic strategies have yet to be widely utilized within the neuro-oncology population, but given their established use in other cancers,^13–15^ may be useful adjuncts for targeting persistent and severe symptoms in a population with high burden.

Other approaches to target symptoms, such as physical activity and exercise, have been encouraged within general oncology, and increasingly so within neuro-oncology patients as well.^16^ For example, Cormie and colleagues (2015) reported the physical and psychological benefits of exercise in patients diagnosed with neurologic malignancies, including reduced fatigue, functional decline, cognitive impairment, anxiety, and depression.^16^ The US Department of Health and Human Services defines physical activity broadly as any body movement that works your muscles and requires more energy than resting, with specific examples including aerobic, muscle strengthening, balance, occupational, and flexibility activities.^17^ There is a wide body of literature supporting the physical and psychological benefits of physical activity and exercise for adults and children,^18^ particularly for those with chronic diseases such as cancer,^19^ but the neuro-oncology population, has unique symptoms that make recommending these types of interventions more challenging. As a result, many patients with brain tumors have persistently low levels of physical activity following their diagnosis,^20^ which may adversely impact their health and well-being.

The purpose of this systematic review is to describe the current landscape and impact of mind-body, cognitive-behavioral, and physical activity interventions for adolescent and adult patients with brain tumors, including both primary and/or secondary (metastatic) brain tumors as both populations suffer from the neurologic and cognitive sequelae of having a tumor in the brain. By querying and synthesizing the existing literature, the authors identify the strengths and limitations of relevant interventions that have been conducted worldwide. Importantly, the authors offer guidance for future research directions that may improve the collective understanding and ability to address the high symptom burden faced by neuro-oncology patients by leveraging non-pharmacological interventions.

## Methods

### Search Strategy

To investigate the published literature related to mind-body, cognitive-behavioral, and physical activity interventions in adolescent and adult neuro-oncology patients, the authors conducted a systematic review according to the preferred reporting items for systematic reviews and meta-analyses (PRISMA) statement checklist.^21,22^ With the assistance of a medical librarian (IDC) and according to the Population, Intervention, Control, and Outcomes (PICO) format,^23^ the Web of Science, Embase, and PubMed databases were queried with the initial search performed on 27 October 2021 with an updated search performed on 21 June 2023. No year limit was set for any of the database searches. Studies were selected according to the following inclusion criteria: (1) involved a mind-body, cognitive-behavioral, or physical activity intervention, (2) included a cohort of patients ≥12 years of age with a primary or secondary (metastatic) brain tumor, (3) reported symptoms/outcomes data, and 4) full-text, published English-language, peer-reviewed, original research. Studies with sample populations that included individuals <12 years of old were considered if they included adolescent and/or adult participants and met other eligibility criteria. Opinion pieces, editorials, commentaries, and review articles were excluded from the review. Additionally, studies with a cohort of <5 brain tumor patients, including case studies, were excluded, given the lack of robust conclusions that could be drawn from such investigations. Backward (ie, snowballing) searches were conducted of reference lists from identified systematic reviews enabling the inclusion of additional relevant reports. However, as outlined previously in the inclusion criteria, systematic reviews themselves were not included as eligible for the present study.

### Diagnoses, Interventions, and Outcomes of Interest

Specific brain tumor diagnoses of interest included glioblastoma, astrocytoma, oligodendroglioma, meningioma, ependymoma, and brain metastases from breast, colon, head and neck, lung, pancreatic, and/or prostate cancer. Mind-body interventions searched for included meditation, yoga, music therapy, and mindfulness while cognitive-behavioral interventions searched for included cognitive therapy, cognitive behavioral therapy, and psychotherapy. Physical activity interventions searched for included exercise, walking, running, jogging, hiking, and training. Lastly, patient outcomes of interest included disease-free survival, fatigue, pain, progression-free survival, recurrence-free survival, sleep, tumor progression, and quality of life. Other tumor diagnoses, non-pharmacologic interventions, and patient outcomes were considered for inclusion if the paper met the inclusion criteria. **Supplementary Table 1** includes a complete listing of the search strategies employed in the present review for each database.

### Article Selection and Data Extraction

**Figure 1.**
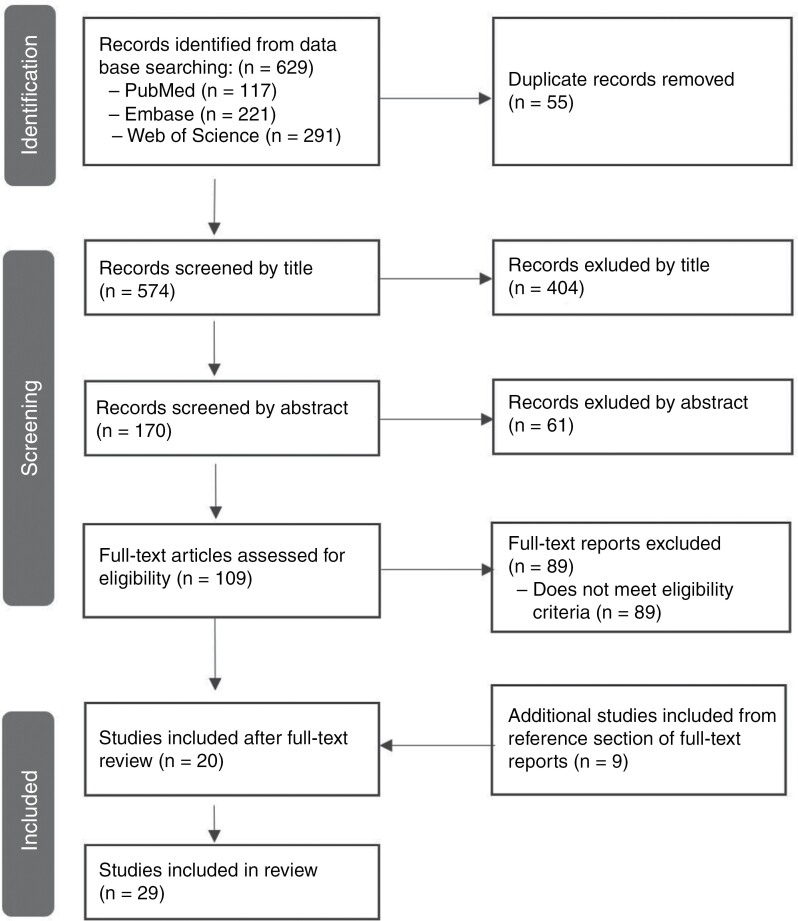
PRISMA flow diagram. A systematic search of 3 databases yielded 629 articles, which was reduced to 20 articles included in the review following duplicate removal and screening for eligibility. An additional 9 studies were identified with the snowballing method from the reference lists of included articles, which yielded a final sample of 29 papers included for systematic review.

The PRISMA flow diagram (**Figure 1**) displays the article selection process employed in this study. IDC performed the database searches identifying a total of 629 papers with 574 articles remaining after duplicate removal. After a title and abstract screen by ARW, 109 full-text articles remained. Two reviewers (ARW and JLR) then independently screened full-text articles according to eligibility criteria and extracted additional articles from reference lists (*n* = 9), ultimately resulting in 29 articles eligible for this review. Disagreements between the 2 independent reviewers were resolved via discussion with senior authors (T.S.A. and A.L.K.). See **Table 1** for details about the studies included in this review.

**Table 1.** Pooled Sample Characteristics for Included Literature

Pooled sample characteristics		
*Geographic location*	# of studies	
North America	12	
South America	1	
Europe	9	
East Asia	3	
Australasia	4	
Total # of participants	1085	
Brain tumors	790	
Non-CNS tumors	205	
Other diseases	15	
Caregivers/relatives	31	
Healthy controls	17	
Not specified^a^	27	
*Age (years)*		
Mean^b^	41	
Range^c^	4–85	
	Count	**%**
*Sex*		
Male	569	52
Female	516	48
*Brain tumor type*		
HGG	121	15
Astrocytoma	94	12
GBM	78	10
Glioma NOS	77	10
Oligodendroglioma	69	9
Meningioma	48	6
Medulloblastoma	32	4
Oligoastrocytoma	20	3
Metastatic	17	2
LGG	15	2
AE	10	1
Pilocytic astrocytoma	6	<1
AA	5	<1
Germ cell tumor	5	<1
Diffuse midline glioma	4	<1
Ependymoma	3	<1
Ganglioma	2	<1
Brain stem glioma	1	<1
Hemangioblastoma	1	<1
Hemangiopericytoma	1	<1
Primary CNS lymphoma	1	<1
Suprasellar teratoma	1	<1
Atypical neurocytoma	1	<1
Retinoblastoma	1	<
Primitive neuroectodermal tumor	1	<1
Metastatic	17	2
Other	35	4
Not specified^d^	124	16

^a^1 study did not specify breakdown of sample population^29^.

^b^1 study did not provide the mean age of included participants^30^.

^c^14 studies did not provide the age range of included participants^27,28,32,34,36-42,47,49,51^.

^d^3 studies did not specify types of brain tumors for participants^29,42,43^.

*Abbreviations:* HGG, high-grade glioma; GBM, glioblastoma; NOS, not otherwise specified; LGG, low-grade glioma; AA, anaplastic astrocytoma; AE, anaplastic ependymoma; CNS, central nervous system.

### Risk of Bias Assessment

Each study’s risk of bias was independently assessed by 3 authors (A.R.W., E.W., and M.M.) based on items from the ROBINS-I (Risk of Bias in Non-Randomized Studies of Interventions) tool. This tool is used to evaluate the risk of bias in estimates of the effectiveness or safety (ie, benefit or harm) of an intervention through the quality assessment of 7 domains, including bias risk related to confounding, selection of participants, classification of the intervention, deviations from the intervention, missing data, outcomes measurement, and results reporting.^24^ Any disagreement in judgments were discussed with senior author A.L.K. and a consensus was reached.

## Results

### Included Studies

The authors identified 29 studies assessing mind-body, cognitive-behavioral, and physical activity interventions conducted in neuro-oncology clinical trials. All 29 studies were prospective trials, including both randomized and non-randomized designs, some of which were single-arm pilot or feasibility studies. Of the 29 interventions, 7 were classified as mind-body interventions,^25–31^ 5 were cognitive-behavioral interventions,^32–36^ and 17 were physical activity interventions.^37–53^ Key study details about the included literature are shown in **Supplemental Table 2**.

### Pooled Sample Characteristics

The 29 included studies generated a pooled sample of 1085 participants, which included a total of 790 brain tumor patients. Studies varied in sample size, with a range of 10 to 150 participants, and in mean age of participants with a range from 4 to 85 years across the literature. Of the 29 included studies, 20 (69%) had brain tumor-only patient populations while 9 (31%) had mixed populations that included brain tumors along with non-CNS tumors,^34,44,45,48^ other diseases not related to cancer,^31,40^ and caregivers or relatives of the participants.^29,30,50^ There were slightly more men (*n* = 569) than women (*n* = 516) who participated in these interventional studies and the most common brain tumor diagnoses represented included high-grade gliomas (*n* = 121, 15%), astrocytoma (*n* = 94, 12%), and glioblastoma (*n* = 78, 10%). Additional details about the pooled sample are shown in **Table 1**.

### Geographical Distribution of Studies

**Figure 2.**
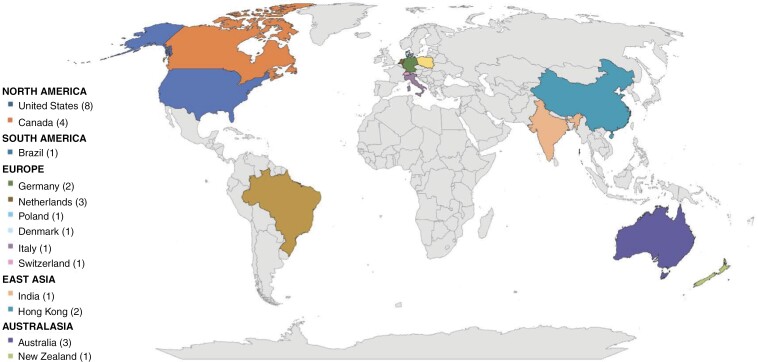
Geographical distribution of included studies. This figure demonstrates the geographical distribution of studies included in this review, including the world region and country. North America had the highest number of studies (12), followed by Europe (9), Australasia (4), East Asia (3), and South America (1). The number of studies from each country is notated in parentheses.

**Figure 2** depicts the geographic distribution of interventional trials included in this review. Most of the trials were conducted in North America (*n* = 12), including 8 studies in the United States and 4 in Canada. Europe was the next most prevalent region for interventional trials including a total of 9 studies in the locations of Denmark (*n* = 1), Germany (*n* = 2), Italy (*n* = 1), the Netherlands (*n* = 3), Poland (*n* = 1), and Switzerland (*n* = 1). The regions of East Asia and Australasia each had 3 interventional studies, which were conducted in Hong Kong (*n* = 2), India (*n* = 1), Australia (*n* = 3), and New Zealand (*n* = 1). Additionally, there was a single interventional study conducted in South America in the country of Brazil.

### Outcomes of Interest and Measurement

Vast heterogeneity was identified regarding the outcomes of interest and instruments used across studies, which made comparing findings across studies difficult and precluded the use of meta-analytic statistical methods. Outcomes of interest in these studies included feasibility (ie, satisfaction, adherence/compliance, accrual, attrition, and safety), physical activity & fitness metrics, neuropsychological performance, cognitive functioning, quality of life, length of stay, amount of medications administered, subjective and objective sleep quality, fatigue, self-esteem, anxiety, distress, depression, symptom severity, and social skills. Quality of life was the only outcome measure that was assessed in the majority of studies (*n* = 18, 62%), though several different instruments were used to measure this symptom. These included the Functional Assessment of Cancer Therapy (FACT-G, -F, and -BR), Medical Outcomes Study 36-item Short-Form survey (SF-36), Chinese version of the Pediatric Quality of Life Inventory Cancer Module v.3.0, Chinese version of the Pediatric Quality of Life Inventory 4.0 Generic Core Scale (PedsQL 4.0), and European Organization for Research and Treatment of Cancer QOL Questionnaire-Core 30 (EORTC QLQ-C30). **Table 1** outlines the instruments utilized in each study and the symptoms or constructs measured.

### Findings Based on Age of Participants

Of the 29 studies included in this review, 19 studies included adult populations (≥18 years of age),^28–30,33–43,49–53^ 7 included pediatric populations (<18 years of age),^25,27,32,44–47^ and 3 studies included a mix of adult and pediatric participants.^26,31,48^ The most common study designs across all age groups were single-arm feasibility trials (12/29 studies), followed by RCTs (10/29 studies), and non-randomized controlled trials (7/29 studies). Among the adult studies, 3 studies included a mind-body intervention,^28–30^ 4 studies included a cognitive-behavioral intervention,^33–36^ and 12 studies included a physical activity intervention.^37–43,49–53^ Nearly all (18/19) of the adult studies reported positive findings for improvement in at least 1 symptom and/or aspect of physical/cognitive functioning, with the most common improvements found in QoL,^29,30,38,42,49,50,52,53^ mood disturbance,^28,30,39,40,42,50,52^ and fatigue.^34,37,39,49,53^ The pediatric studies included 2 mind-body interventions,^25,27^ 1 cognitive behavioral intervention,^32^ and 4 studies with physical activity interventions.^44–47^ All of the pediatric literature reported positive findings with the most common improvements found in QoL,^25,32,44,45^ and fatigue.^27,44,46^ Lastly, there were 3 mixed sample populations with respect to age, all of which reported positive findings with the most common improvements in QOL^26,31^ and mood disturbance.^31^

### Mind-Body Interventions

#### Intervention details

Of the 29 studies identified, 7 (24%) tested mind-body interventions in a total of 200 participants. There were several types of mind-body interventions represented in the literature, including yoga,^27,29,30^ music therapy,^25,31^ massage therapy,^28^ and app-based mindfulness training.^26^ There were 2 dyadic yoga studies that included newly diagnosed high-grade glioma patients on active treatment and their caregivers in a Vivekananda yoga program led by certified instructors.^29,30^ The remaining yoga study was conducted in patients who were on active treatment with the intervention delivered by a certified yoga instructor with parents also present for the sessions.^27^ Additionally, there were 2 studies that focused on music therapy as an intervention,^25,54^ with one providing live music sessions to patients in the perioperative period, while the other trained patients who were on imaging surveillance to play a musical instrument. The remaining 2 mind-body interventions included a massage therapy program for newly diagnosed patients^28^ and mindfulness training using an app-based program for patients with primary and metastatic brain tumors.^26^

For the mind-body intervention studies, there was no standard follow-up duration, frequency, or length for the assessments and interventions. Post-intervention follow-up periods ranged anywhere from 1 to 12 weeks, with the majority of studies assessing intervention efficacy at more than 1 timepoint (*n* = 6).^25,26,28–31^ All studies recommended the use of the mind-body interventions at least 1 day per week with most study designs mandating sessions 2 to 3 times per week. The prescribed length of interventions varied depending on the type of mind-body intervention, with yoga sessions between 45 and 60 minutes (if specified),^29,30^ music therapy between 20 and 45 minutes,^25,31^ massage sessions were 45 minutes,^28^ and a minimum of 10 minutes for the mindfulness app intervention.^26^

#### Main findings

The main outcomes assessed across the mind-body literature included feasibility, mood disturbance (including distress, anxiety, and/or depressive symptoms), and quality of life. Of the 7 mind-body studies, 4 considered the feasibility of their interventional approaches,^26,27,29,30^ based on adherence and retention rates, rate of adverse events (AEs), and participant satisfaction, with 3 studies (all yoga interventions) reporting high feasibility.^27,29,30^ De Tommassi et al. reported high satisfaction with their mindfulness app-based intervention, but they had significant attrition from the trial which adversely impacted their feasibility. The most commonly measured patient-reported outcomes in the mind-body literature were related to mood disturbance, including depression, anxiety, and stress symptoms, which were measured in 6 of the 7 mind-body studies.^25,26,28–31^ Music therapy, yoga, and massage therapy interventions all showed global improvements in mood, while the mindfulness app-based intervention did not result in the reduction of anxiety or depressive symptoms.^26^ Several mind-body trials also measured quality of life, which improved post-intervention for 4 of the 5 studies that measured this variable, which were music therapy and yoga trials.^25,29–31^ The 2 trials by Milbury et al.^29,30^ were unique in that they had a dyadic interventional design for patients and their caregivers to complete a 12-week yoga program while the patient received radiation therapy, with both parties reporting improved quality of life at the end of the intervention period.

### Cognitive-Behavioral Interventions

#### Intervention details

Cognitive-behavioral interventions were the least frequently utilized approach in the included neuro-oncology literature (*n* = 5, 17%), which included a total of 241 patients (half of which were from a single study^34^). There was significant heterogeneity of interventional approach across these 5 trials, including a social skills building program,^32^ an online self-help depression program,^34^ an educational fatigue program,^33^ goal management cognitive training,^35^ and cognitive rehabilitation,^36^ though they all aimed to improve a particular symptom or functional parameter. There were 2 studies with cognitive interventions that were administered by trained psychologists, including goal management training for patients at least 3 months out from treatment^35^ and cognitive rehabilitation for patients less than 2 weeks out from cranial surgery.^36^ Boele et al.^34^ reported findings from a large RCT that utilized an online course based on problem-solving therapy for patients in various phases of treatment. The remaining cognitive-behavioral studies included a supervised group social skills intervention for patients who were in treatment follow-up,^32^ as well as a leaflet-based educational approach for fatigue management for patients undergoing chemoradiation.^33^

For the cognitive-behavioral studies, there was no standard follow-up duration, frequency, or length for the assessments and interventions. Post-intervention follow-up periods ranged anywhere from 1 week to 12 months, with the majority of studies measuring intervention efficacy at more than 1 timepoint (*n* = 3),^33–35^ often within 3 to 6 months of intervention completion. All studies included interventions that were supervised by psychology-trained individuals, including postdoctoral students, online coaches, and psychologists, in order to maximize intervention fidelity. The prescribed frequency and length of interventions varied widely depending on the methodological approach, with session frequencies ranging from 1 to 4 days per week for the more structured interventions with the duration of interventions typically between 4 and 12 weeks. In the trial by dos Reis Bigatao,^33^ their intervention was less structured with no recurring sessions following provision of the educational materials focused on non-pharmacological fatigue management.

#### Main findings

The most common outcomes assessed across these cognitive-behavioral intervention studies included feasibility, depression, fatigue, cognitive function, and quality of life. Feasibility was assessed in 3 of the 5 studies with mixed results. Barrera et al.^32^ and Richard et al.^35^ both reported high feasibility for their trials with minimal attrition and high intervention adherence, while Boele et al. reported high levels of attrition with only 50% of participants completing the 8-week follow-up assessments and 15% completing the 12-week follow-up assessments.^34^ Depression was assessed following interventions in 3 cognitive-behavioral trials,^32–34^ all of which reported no significant improvements for the intervention groups. Fatigue had mixed results with improvement following an online self-help intervention for depression,^34^ yet an educational leaflet intervention focused on fatigue management did not yield significant improvements.^33^ Only 2 studies had cognitive-behavioral interventions targeting various aspects of cognitive function,^35,36^ including executive functioning, memory, and attention, with both trials reporting significant improvements for patients post-intervention. Lastly, there were 3 cognitive-behavioral trials that measured quality of life as an outcome measure, which only improved following a group social skills-building intervention for childhood brain tumor survivors.^32^

### Physical Activity Interventions

#### Intervention details

More than half (*n* = 17, 59%) of the studies identified utilized physical activity interventions, testing such interventions on 644 total participants. While the types of physical activities differed across interventions, all studies included exercise programs that implemented some component of aerobic training, which aimed to improve cardiovascular health by performing actions that increase breathing and heart rates. Aerobic training was performed by using stationary bikes, treadmills, prolonged walking, and skiing. Additionally, 6 of the 16 physical activity studies included resistance training using exercise bands or weightlifting,^38,39,44,46,48,49^ which are intended to strengthen specific muscle groups and improve physical fitness. Additionally, Hansen et al.^53^ incorporated an occupational therapy intervention alongside the physical exercise intervention for individuals who identified needs in this area, including self-care, productivity, or other activities of daily living. The timing of when physical activity interventions were conducted across the literature varied, with several trials focusing on patients who have completed treatment and are on imaging surveillance,^40–43,45,47,48,52^ while others included patients on active treatment^38,39,44,46,49–51^ and in the postoperative period.^37,38,53^

For studies testing physical activity interventions, similar to the mind-body and cognitive-behavioral interventions, there was no standard follow-up duration, frequency, or length for the assessments and interventions. Intervention durations ranged from 1 to 31 week(s), with 15 of the 16 physical activity intervention studies lasting ≥4 weeks.^37–49,52,53^ Notably, 12 weeks was the most common length for physical activity interventions (*n* = 5).^39,40,43,46,47^ Studies differed in the number of interventions offered throughout the study duration with the majority of study designs requiring participants to complete a defined number of sessions per week^37–43,46–53^ and only 1 study not specifying a session number requirement.^45^ Another trial included a highly intense, 28-session intervention that was completed over a 31-week period.^44^ There was no common theme for the length of intervention sessions in the physical activity studies, including some with a set time limit, others with no time limit, and some not specifying if sessions were timed. Those studies with a timed intervention session ranged from 20 minutes to 4 hours,^40,43,44,47,49–53^ with the majority offering intervention sessions >30 minutes. Additionally, 3 of the 16 physical activity studies had no time limit for their respective intervention sessions^39,46,48^ and participants were told to complete the regimen at their own pace. Lastly, 4 of the 16 physical activity studies did not specify the length of their intervention sessions.^38,41,42,45^

#### Main findings

The primary outcomes assessed in these physical activity intervention studies included feasibility, physical fitness, quality of life, and psychological functioning. Importantly, 14 of the 17 physical activity intervention studies tested the feasibility of the intervention, which was most often assessed via study attrition, adherence to the prescribed intervention, and AEs. Among these feasibility studies, most reported that the intervention techniques investigated were indeed feasible in their samples with minimal attrition, good adherence, and no intervention-related adverse events (AEs) reported. Of the 3 studies that had poorer feasibility metrics,^48,51^ the most common reasons were related to the impact of treatment (ie, prohibitive side effects) and a perceived lack of time to dedicate to the exercise intervention.

Another common outcome for the physical activity intervention studies was physical fitness, which was measured in 13 of the 17 studies taking into account the participants’ fitness before and after the intervention.^37,39,41,44–53^ Fitness was most often measured by assessing changes in cardiorespiratory fitness, exercise endurance, hand-grip strength, and steps taken per day, among other physiological parameters. While the majority of these studies reported improvements in participant fitness after their respective interventions, 2 studies reported no change in fitness, though they were likely underpowered and had issues with intervention fidelity and consistency.^46,48^

Lastly, 10 of the 17 physical activity intervention studies assessed changes in quality of life^38,40,42,44–46,49,50,53^ and psychological functioning^40,41,43,48^ as outcome measures with promising findings across the literature. Quality of life, which was assessed by measuring fatigue, functional status, and sleep disturbances, improved following a physical activity intervention in 8 of 9 studies that measured this patient-reported outcome. Psychological functioning was most often assessed by measures of depression, cognition, distress, and anxiety and showed global improvement in all 4 studies that assessed these variables following a physical activity intervention.

### Risk of Bias Assessment

**Supplementary Table 3** shows a detailed risk of bias assessment for the literature in this review, including domain-based judgments as well as an overall bias judgment for each study. Based on these assessments, all studies were at moderate to high risk of bias with the most concerning study aspects relating to a lack of control for confounding variables and a significant amount of missing data, typically related to study attrition. Moderate to severe risk of bias was reported in 18 studies related to confounding (with 3 serious ratings)^26,31,37^ and in 17 studies related to missing data (with 5 serious ratings).^26,38,43,45,51^ Of note, 20 of 29 studies had a low risk of bias related to the classification of the intervention due to generally very detailed methodological descriptions of the interventions delivered to participants.

## Discussion

This systematic review included 29 studies assessing the feasibility and efficacy of mind-body, cognitive-behavioral, and physical activity interventions to improve symptoms and quality of life for adults and children with brain tumors. There was a great deal of variation in the types of interventions utilized, the intervention dose (ie, frequency and duration of sessions), and the outcome variables of interest and how they were measured. Still, efficacy results were promising with all but 1 study^33^ reporting significant improvement in at least 1 aspect of physical, cognitive, and/or psychological functioning after the intervention. While there is extensive literature surrounding the use of non-pharmacological interventions in other cancer populations,^55–57^ there is a dearth of evidence specific to such interventions within the brain tumor population and this review aimed to fill that gap.

A recent systematic review and meta-analysis by Cillessen et al.^57^ demonstrated that mind-body interventions are efficacious in reducing the severity of physical and psychological symptoms for cancer survivors, yet many of the reported effect sizes have been modest to date. Patients with brain tumors have been shown to have higher mood disturbance compared to other cancer populations,^58^ which highlights the need for non-pharmacological interventions to address these symptoms before they become more pervasive psychological disorders that are difficult to treat. There were 7 mind-body interventions represented in this review, including yoga, music therapy, massage, and the use of a mindfulness-based app, the majority of which did show significant improvements in mood and quality of life, though the duration of these effects was not robustly tested due to short follow-up periods. Additionally, several of these studies had single-arm designs with small sample sizes, which may inflate efficacy metrics. With regards to the feasibility of these approaches, retention and adherence rates were high, particularly in trials where the intervention was supervised by a trained instructor^27,29,30^ compared to self-guided interventions.^26^ This advantage for intervention supervision has been reported in the broader cancer literature using these approaches,^59,60^ but raises an important question of feasibility for neuro-oncology patients who may not have logistical or financial access to trained mind-body specialists. It may be prudent in future trials to have upfront instruction on mind-body techniques by trained individuals to help patients learn those strategies and then transition to self-guided therapy over time, which would promote self-efficacy and increase feasibility for the broader population.

Cognitive-behavioral therapy is a psychotherapeutic approach that has a long history of use within oncology to help address the various psychological symptoms that patients with cancer experience throughout their illness trajectory. It is centered on the premise that it is difficult to change our emotions and related symptoms directly, so the goal of this approach is to first address problematic thoughts and behaviors in order to see improvement in how we feel.^12^ In our review, we had 5 different studies utilizing very different types of CBT, including a social skills-building group intervention, an online self-help program for depression, an educational intervention targeting fatigue, and 2 different cognitive training interventions, the majority of which were supervised by trained psychology personnel and involved several sessions per week for at least 1 month. Despite most studies utilizing randomized controlled trial designs, efficacy was minimal for improving symptoms such as depression and fatigue,^32–34^ though the interventions targeting cognition did report improvements in executive functioning, memory, and attention.^35,36^ There are several factors that likely contributed to lackluster efficacy findings in these cognitive-behavioral intervention trials, including small samples and high attrition that contributed to underpowered statistical analyses. One key disadvantage to cognitive-behavior approaches is the significant time commitment that is often involved with treatment durations of up to 12 months in some cases.^34^ Additionally, similar to the mind-body interventions described above, the majority of the reviewed cognitive-behavioral interventions required direct supervision throughout the treatment period by a trained specialist, which is not always feasible or practical for cancer patients. In this post-COVID era where telehealth platforms have been leveraged to provide remote care, few trials have tested virtually administered cognitive-behavioral interventions for cancer patients, which may be just as effective as in-person therapy and could alleviate some of the perceived burden for the individual receiving treatment.

Impaired physical functioning is a common ramification of cancer and its treatment, which can adversely impact functional independence, tolerability of anti-cancer treatments, and disease-free and overall survival.^19,61^ Past research has provided strong evidence that physical activity is feasible and safe for patients to do at any stage of the illness trajectory and across all cancer types, including those with advanced stages of disease, and patients tend to experience improvements in physical and psychological health.^55^ Our findings aligned with these past data with the vast majority of physical activity trials in this review reporting their interventions as feasible with good adherence, minimal attrition from the study, and few AEs that could be attributed to the intervention. Of the 3 trials that reported a lack of intervention feasibility, the reasons cited included poor adherence to the prescribed exercise dosage,^51^ high attrition as patients transitioned from inpatient to outpatient sessions,^37^ and a high proportion of patients refusing follow-up assessments after the intervention was complete.^40^ Regardless of the setting of the intervention (ie, inpatient vs. outpatient), interventions that incorporated some degree of individualized training with specialists like physical therapists or exercise physiologists tended to have the best adherence and attrition metrics.^37,41,42^ This suggests that patients may benefit more from individual, supervised sessions compared to interventions delivered in a group setting, which is similar to what we saw with the mind-body and cognitive-behavioral interventional strategies.

Physical activity and exercise have strong evidence and a sound theoretical basis for their effect on reducing symptom burden, improving physical functioning, and enhancing quality of life.^19^ Studies included in this review reported largely positive efficacy data for various kinds of physical activity interventions to improve physical fitness and strength, as well as improvements in fatigue, sleep, drowsiness, mood, and quality of life, which aligns with previously published work.^55^ Studies in this review that reported no improvement in symptoms or other patient-reported outcomes tended to be smaller feasibility trials with significant attrition or other methodological issues.^48,51^ There have been somewhat mixed efficacy results for physical activity interventions reported in past research with disparities in findings typically attributed to heterogeneous study designs and measurement approaches.^55^ The American College of Sports Medicine recommends that all cancer survivors should aim to participate in at least 150 minutes of moderate exercise (ie, brisk walking, swimming) or 75 minutes of vigorous exercise (ie, jogging, running) each week.^62^ However, patients with brain tumors may lack both the knowledge and the confidence on how to safely exercise to meet these prescribed physical activity goals. Additionally, there may be reluctance on the part of their clinicians to encourage an exercise regimen given the typically poor prognosis and high symptom burden in this patient population.^19^ The American Cancer Society has emphasized the importance of recognizing that symptoms may be a barrier to the adoption of healthy behaviors for patients,^63^ which highlights the importance of aggressive symptom management for patients from the time of diagnosis extending into survivorship. This approach may allow patients to adopt or maintain healthy physical activity behaviors earlier in their illness trajectory, which may improve their overall health and prolong survival.

While the majority of included literature focused on adult neuro-oncology patients, there were a few studies that were conducted exclusively among children (<18 years of age) or had mixed samples that included both adults and children. There is growing support among oncology researchers and from regulatory agencies, such as the Food and Drug Administration, to consider inclusion of adolescents (12 to 17 years) in cancer clinical trials of all kinds due to the fact that this group has been consistently overlooked in research and they have unique needs that may differ from their adult counterparts,^8,9^ which applies to the neuro-oncology population as well.^64^ This review sought to include younger patients to try and address this gap, though we recognize that some younger children existed in some of those samples, which may have biased or diluted efficacy findings. We reported on 7 studies that included pediatric patients utilizing mind-body, cognitive-behavioral, and physical activity interventions with largely positive efficacy results for improvements in quality of life, mood disturbance, and fatigue. There have been recent recommendations to incorporate age-appropriate mindfulness-based interventions within pediatric oncology, including approaches such as body scans, mindful breathing, and mindfulness movements, which have been shown to improve symptoms such as fatigue, anxiety, sadness, and anger in children with various types of cancer.^65^ There is also growing interest in virtual reality-based mindfulness approaches that show promise for targeting a variety of symptoms for patients with cancer,^66,67^ which could be tailored to the age and developmental stage of the patient. Further interventional research is needed in pediatric neuro-oncology to ensure we are meeting the needs of children with cancer and their families so that their quality of life can be optimized.

### Future Directions

While findings from this review are promising for the feasibility and efficacy of mind-body, cognitive-behavioral, and physical activity interventions for neuro-oncology patients, there is a need for larger, more robustly designed RCTs to definitively assess potential benefits and risks of these approaches in this understudied population. In addition to stronger study designs, it would benefit the field to have multi-center trials that would allow more diverse patients to participate in these studies. Particular attention should be paid to recruiting underrepresented groups within neuro-oncology so that there is a better representation of intervention effects for those individuals in order to promote better generalizability. Longitudinal assessment of symptoms would also allow assessment of longer-term for these non-pharmacologic interventions, which is something that is severely lacking in the literature to date. The use of smart wearables and remote symptom monitoring applications could be ideal tools to incorporate into future trial designs to allow for such data to be collected in a systematic way under the guidance of trained clinicians and researchers to ensure data fidelity.

### Study Limitations

This review had several limitations that are important to acknowledge. The significant heterogeneity of the included literature in terms of sample characteristics, interventions, outcomes of interest, and outcomes measurement makes drawing definitive conclusions about the strength of the evidence challenging and limits our ability to perform a meta-analysis. Additionally, while our aim was to target interventions that target adolescent and adult patients with CNS tumors, some papers included children younger than 12 years of age in their sample, which may have impacted findings, particularly if that approach is better suited for older individuals. There were also studies with mixed tumor samples that did not report results separately for PBT patients apart from metastatic brain tumors or other types of cancer, which may have impacted findings. Lastly, several studies included in the review were single-arm feasibility or pilot trials that had relatively small sample sizes and no true control group, therefore those results should be interpreted with caution and necessitate validation in larger, more rigorously designed trials. Nevertheless, our findings are compelling and underscore the need for additional research into these interventional strategies in order to improve patient outcomes within neuro-oncology.

## Conclusion

This review highlights the feasibility and efficacy of mind-body, cognitive-behavioral, and physical activity interventions for adolescent and adult patients with brain tumors, with the majority of included studies showing improvement in physical and/or psychological outcomes. In a highly symptomatic patient population with an often dismal prognosis, it is of the utmost importance to prioritize maximizing their quality of life and promoting healthy behaviors and strategies so that they can better self-manage symptoms and optimize their health.

## Supplementary material

Supplementary material is available online at *Neuro-Oncology* (https://academic.oup.com/neuro-oncology).

vdae134_suppl_Supplementary_Table1

vdae134_suppl_Supplementary_Table2

vdae134_suppl_Supplementary_Table3

## Data Availability

Data sharing is not applicable to this article as no new data were created or analyzed in this study.
