# Supplementary material for: Landscape and impact of mind-body, cognitive-behavioral, and physical activity interventions in adolescent and adult brain tumor patients: A systematic review
Source: Neurooncol Adv. 2024 Aug 22;6(1):vdae134. doi: 10.1093/noajnl/vdae134 (PMC11445902; doi:10.1093/noajnl/vdae134)
Supplement: vdae134_suppl_Supplementary_Table1 [file vdae134_suppl_supplementary_table1.docx]

**Supplementary Table 1. Search strategies and citation counts**

| *I. Initial databases search on 27 October 2021, updated search on 30 June 2023*  ***Web of Science = 291 citations***  TOPIC: (Brain neoplasms OR primary brain tumor OR primary brain tumors OR secondary brain tumor OR secondary brain tumors OR secondary brain cancer OR secondary brain cancers OR brain metastases OR malignant brain neoplasm OR malignant brain neoplasms OR brain cancer OR brain cancers OR brain malignant neoplasm OR brain malignant neoplasms OR intracranial neoplasm OR intracranial neoplasms OR glioma OR Glioblastoma OR Astrocytoma OR Oligodendroglioma OR Meningioma OR meningioma OR Ependymoma) AND TOPIC: (Meditation OR Cognitive therapy OR cognitive behavioral therapy OR Exercise OR Physical activity OR physical exercise OR aerobic exercise OR physical activities OR Walking OR Jogging OR running OR Hiking OR Yoga OR Mindfulness OR Mind body therapies OR Complementary therapies OR Complementary therapy OR Swimming OR stair climbing) AND TOPIC: (Disease-free survival OR Progression-free survival OR progression free survival OR symptom reduction OR Improved quality of life OR quality of life OR Sleep OR Fatigue OR Pain OR Tumor progression OR Anxiety OR Depression OR psychological stress) |
| --- |
| ***Embase = 221 citations***  ('brain neoplasms'/exp OR 'brain neoplasms' OR 'primary brain tumor'/exp OR 'primary brain tumor' OR 'primary brain tumors' OR 'secondary brain tumor' OR 'secondary brain tumors' OR 'secondary brain cancer' OR 'secondary brain cancers' OR 'brain metastases' OR 'malignant brain neoplasm' OR 'malignant brain neoplasms' OR 'brain cancer'/exp OR 'brain cancer' OR 'brain cancers' OR 'brain malignant neoplasm' OR 'brain malignant neoplasms' OR 'intracranial neoplasm'/exp OR 'intracranial neoplasm' OR 'intracranial neoplasms' OR 'glioma'/exp OR glioma OR 'glioblastoma'/exp OR glioblastoma OR 'astrocytoma'/exp OR astrocytoma OR 'oligodendroglioma'/exp OR oligodendroglioma OR 'meningioma'/exp OR meningioma OR 'ependymoma'/exp OR ependymoma) AND ('meditation'/exp OR meditation OR 'cognitive therapy'/exp OR 'cognitive therapy' OR 'cognitive behavioral therapy'/exp OR 'cognitive behavioral therapy' OR 'exercise'/exp OR exercise OR 'physical activity'/exp OR 'physical activity' OR 'physical exercise'/exp OR 'physical exercise' OR 'aerobic exercise'/exp OR 'aerobic exercise' OR 'physical activities' OR 'walking'/exp OR walking OR 'jogging'/exp OR jogging OR 'running'/exp OR running OR 'hiking'/exp OR hiking OR 'yoga'/exp OR yoga OR 'mindfulness'/exp OR mindfulness OR 'mind body therapies'/exp OR 'mind body therapies' OR 'complementary therapies'/exp OR 'complementary therapies' OR 'complementary therapy' OR 'swimming'/exp OR swimming OR 'stair climbing'/exp OR 'stair climbing') AND ('disease-free survival'/exp OR 'disease-free survival' OR 'progression-free survival'/exp OR 'progression-free survival' OR 'progression free survival'/exp OR 'progression free survival' OR 'symptom reduction' OR 'improved quality of life' OR 'quality of life'/exp OR 'quality of life' OR 'sleep'/exp OR sleep OR 'fatigue'/exp OR fatigue OR 'pain'/exp OR pain OR 'tumor progression'/exp OR 'tumor progression' OR 'anxiety'/exp OR anxiety OR 'depression'/exp OR depression OR 'psychological stress'/exp OR 'psychological stress') |
| ***PubMed = 117 citations***  (Brain neoplasms[Majr] OR brain neoplasm*[tiab] OR primary brain tumor*[tiab] OR secondary brain tumor*[tiab] OR secondary brain cancer*[tiab] OR brain metastases[tiab] OR malignant brain neoplasm*[tiab] OR brain cancer*[tiab] OR brain malignant neoplasm*[tiab] OR intracranial neoplasm[tiab] OR glioma[Mesh] OR glioma[tiab] OR Glioblastoma[Majr] OR glioblastoma[tiab] OR Astrocytoma[Mesh] OR astrocytoma[tiab] OR Oligodendroglioma[Mesh] OR oligodendroglioma[tiab] OR Meningioma[Mesh] OR meningioma[tiab] OR Ependymoma [Mesh] OR ependymoma[tiab]) AND (Meditation[Mesh] OR Meditation[tiab] OR Cognitive therapy[tiab] OR cognitive behavioral therapy[Mesh] OR Exercise[Majr] OR exercise[tiab] OR Physical activity[tiab] OR physical exercise[tiab] OR aerobic exercise[tiab] OR physical activities[tiab] OR Walking[Mesh] OR Walking[tiab] OR Jogging[tiab] OR running[tiab] OR Hiking[tiab] OR Yoga[Mesh] OR yoga[tiab] OR Mindfulness[Mesh] OR mindful*[tiab] OR Mind-body therapies[Mesh] OR mind body therap*[tiab] OR Complementary therapies[Mesh] OR Complementary therapy[tiab] OR Swimming[tiab] OR stair climbing[tiab]) AND    (Disease-free survival[Mesh] OR disease-free survival[tiab] OR disease free survival[tiab] OR Progression-free survival[Mesh] OR progression-free survival[ti] OR progression free survival[ti] OR symptom reduction[tiab] OR Improved quality of life[tiab] OR quality of life[tiab] OR Sleep[Mesh] OR sleep[tiab] OR Fatigue[Mesh] OR fatigue[tiab] OR Pain[Mesh] OR Pain[tiab] OR Tumor progression[tiab] OR Anxiety[Mesh] OR anxiety[tiab] OR Depression[Mesh] OR depressi*[tiab] OR psychological stress[tiab] OR stress, psychological[Mesh]) |
| *II. Citations counts*  Total citations from all three databases = **629**  Duplicate citations removed = **55**  Total citation results for review = **574** |
