# Supplementary material for: Landscape and impact of mind-body, cognitive-behavioral, and physical activity interventions in adolescent and adult brain tumor patients: A systematic review
Source: Neurooncol Adv. 2024 Aug 22;6(1):vdae134. doi: 10.1093/noajnl/vdae134 (PMC11445902; doi:10.1093/noajnl/vdae134)
Supplement: vdae134_suppl_Supplementary_Table2 [file vdae134_suppl_supplementary_table2.docx]

**Supplementary Table 2. Data extraction table for included literature**

| **Author (Year)** | **Population & Sample** | **Primary Objective** | **Study Design & Outcomes** | **Key Findings & Limitations** |
| --- | --- | --- | --- | --- |
| ***MIND-BODY INTERVENTIONS*** | |  |  |  |
| Cheung (2019)^23^ | 60 participants  Ages 7 to 16 years (mean 13)   - Majority male - Individuals in remote (home) setting with reported depressive symptoms   Unspecified brain tumors  East Asia (Hong Kong) | To examine the efficacy of a musical training program in reducing depressive symptoms & enhancing self-esteem and QoL in pediatric brain tumor survivors | **Design:**   - RCT, between-subject design   - 2 study arms (intervention group = music training, control group = placebo intervention)   - Timing: 2+ months after completion of treatment - Training with patients done by orchestral performers at participants’ homes (one-to-one lessons)   - 45 min music intervention (1 day/week) for 1 year   - Placebo group also received weekly visits of same duration but engaged in unstructured activities (unrelated to music)   **Outcomes:**   - Depressive symptoms (CES-DC) - Self-esteem (RSES) - QoL (PedsQL 4.0) | **Findings:**   - Experimental group reported significantly fewer depressive symptoms (*p*<.001), higher self-esteem (*p*<.001) & better QoL (*p*<.001) at 12-month follow-up   **Limitations:**   - No true control group (no home visits) - Power analysis not conducted |
| De Tommasi (2020)^24^ | 20 participants  Ages 16 to 74 years (mean 54)   - Majority female - Individuals with recent cranial surgery   Primary & metastatic brain tumors  Australasia (New Zealand) | To determine the feasibility of an 8-week mindfulness intervention by app-based training (ABT) in patients with brain tumors | **Design:**   - Feasibility trial   - Single arm (no control group)   - Timing: during treatment - Mindfulness ABT intervention done at home for 8 weeks   **Outcomes:**   - Feasibility (4-item measure of satisfaction & Headspace data) - Anxiety/Depression (HADS) - HRQoL (FACT-Br, SF-12) - Mindfulness capacity (FFMQ) | **Findings:**   - 65% of participants used the app regularly & most found it useful - No significant differences in anxiety or depression post-intervention - Significant improvement in mindfulness capacity related to observing and being less reactive (*p*=.07) - Considerable attrition   **Limitations:**   - Lack of control group - Small sample size - Significant attrition - Use of paper questionnaires was burdensome for participants |
| Govardhan (2019)^25^ | 18 participants  Ages 6 to 18 years (mean 10)   - Even gender distribution - Individuals in outpatient setting who are undergoing treatment for a brain tumor   Primary brain tumors  East Asia (India) | To establish the feasibility & therapeutic effect of yoga in pediatric brain tumor patients and to provide a foundation for development of an RCT | **Design:**   - Feasibility trial   - Single study arm   - Timing: during treatment - Yoga interventions with patients done by licensed yoga instructor   - Patients required to complete minimum of 3 days/week for 4 weeks (parent/guardian required to be present during sessions)   **Outcomes:**   - Feasibility (adherence, attendance, satisfaction) - Symptoms (parent proxy-reported form) | **Findings:**   - All participants achieved minimum 3 yoga sessions per week (median # of sessions=16) - Parents reported that children looked forward to the sessions as an ideal opportunity to unwind - Significant improvements in participant pain (*p*=.0002), headaches (*p*=.0005), appetite (*p*=.0005), sleep (*p*=.0003) & fatigue (*p*=.007) - Increase in overall daily activity was also significant (*p*=.0018) - No AEs attributed to yoga were reported   **Limitations:**   - Lack of control group - Small sample size |
| Keir (2012)^26^ | 25 participants  Ages 24 to 70 years (mean 49)   - Majority male - Individuals in outpatient setting who were newly diagnosed with a brain tumor & had reported distress   Primary brain tumors  North America (United States) | To obtain a preliminary assessment of the effect of massage therapy on patient-reported psychological outcomes & QoL | **Design:**   - Pilot trial   - Single study arm   - Timing: during treatment - 45-min massage intervention (2 days/week) by licensed massage therapists for 4 weeks (total of 8 sessions)   **Outcomes:**   - Distress (DT) | **Findings:**   - Significant improvement in distress levels between baseline & week 3 (*p*≤.025) and further reductions between weeks 3 & 4 (*p*≤.001) - Significant predictors of distress included worry (β=.337, *p*<.05), sadness (β=.317, *p*<.05), nervousness (β=.307, *p*<.05) & pain (β=.298, *p*<.05) - Patient-reported items of concern significantly dropped in # from baseline to end of week 3 (*p*<.001), week 4 (*p*<.001) & week 5 (*p*<.001)   **Limitations:**   - Lack of control group - Small sample size |
| Milbury (2018)^27^ | 10 participants  Mean age 55 years   - Majority female - Individuals & their caregivers in outpatient setting who were newly diagnosed with a high-grade glioma & are undergoing radiation   Primary brain tumors  North America (United States) | To establish feasibility of a patient-caregiver dyadic yoga program (DYP) for newly diagnosed NGG patients & their family caregivers targeting QoL outcomes | **Design:**   - Pilot trial   - Single study arm   - Timing: during treatment - Training with patients done by a certified yoga instructor   - Breathing exercises, gentle movements & guided meditations - Dyads attended 2-3 weekly sessions (60 min each) for 5-6 weeks (12 sessions total)   **Outcomes:**   - Feasibility (attendance, adherence, attrition) - Symptoms & interference (MDASI) - Depressive symptoms (CES-D) - Sleep disturbance (PSQI) - Fatigue (BFI) - QoL (SF-36) | **Findings:**   - All 5 dyads completed the 12 DYP session with 67% rating program as beneficial - Significant improvements in patient-reported symptom burden (*d*=1.03), sleep (*d*=1.17) & QoL (*d*=.6) - Caregivers reported significant improvement in patients’ symptom burden (*d*=.67) & their own QoL (*d*=.64) - Worsening of caregiver depressive symptoms reported (*d*=1.04)   **Limitations:**   - Lack of control group - Small sample size - High performing participants at baseline (ceiling effect) |
| Milbury (2019)^28^ | 40 participants  Mean age 55 years   - Majority female - Individuals & their caregivers in outpatient setting who are undergoing chemotherapy treatment   Primary brain tumors  North America (United States) | To examine the feasibility & preliminary efficacy of a dyadic yoga (DY) intervention as a supportive care strategy | **Design:**   - Pilot RCT   - 2 study arms (intervention group = yoga, control group = usual care)   - Timing: during treatment - Training with patients done by certified yoga instructors - 45 min yoga sessions (2-3 days/week) for 4-5 weeks (12 sessions total)   **Outcomes:**   - Feasibility (adherence, retention) - Cancer-related symptoms (MDASI-BT) - Depressive symptoms (CES-D) - Fatigue (BFI) - QoL (SF-36) | **Findings:**   - High adherence (88%) & retention (95%) rates - Clinically significant improvements for patients related to symptom severity (*d*=.96), symptom interference (*d*=.74), depressive symptoms (*d*=.71) & mental QoL (*d*=.69) - Caregivers reported clinically significant improvements in depressive symptoms (*d*=1.12), fatigue (*d*=.89) & mental QoL (*d*=.49)   **Limitations:**   - Small sample size - High performing participants at baseline (ceiling effect) |
| Walworth (2008)^29^ | 27 participants  Ages 8 to 73 years (mean 47)   - Primarily female - Individuals in inpatient setting who were receiving an elective brain surgery   Tumor types not specified (mixed sample with non-tumor diagnoses)  North America (United States) | To examine the effects of live music therapy on QoL indicators, amount of medications administered & length of stay for those receiving elective surgical procedures of the brain | **Design:**   - RCT   - 2 study arms (intervention group = music, control group = no music)   - Timing: pre- and post-operative period - 20-30 min music therapy intervention in pre-op room, repeated sessions each day of their hospitalization   - Pre/post assessments surrounding each session   **Outcomes:**   - QoL indicators [anxiety, mood, pain perception of hospitalization, relaxation, stress] (VAS) - Pain/nausea medications administered (frequency, dose, type, route) - Hospital LOS (time of admission to discharge) | **Findings:**   - Significant improvements reported for anxiety (*p*=.03), perception of hospitalization (*p*=.03), relaxation (*p*=.001) & stress (*p*=.001) - No significant improvements in pain or mood - Too few participants received medications to treat pain and/or nausea, so no statistical comparisons made   **Limitations:**   - Small sample size - Variation in interventions delivered due to busy hospital environment - Session lengths varied |
| ***COGNITIVE-BEHAVIORAL INTERVENTIONS*** | | |  |  |
| Barrera (2009)^30^ | 32 participants  Ages 8 to 18 years   - Majority male - Individuals in outpatient setting who were on long-term follow up   Primary brain tumors  North America (Canada) | To evaluate the feasibility & preliminary outcomes of a social skills group intervention program for child brain tumor survivors | **Design:**   - Feasibility trial, repeated measures design   - Single study arm (each participant was his/her own control)   - Timing: in follow-up after treatment completion - Training with patients done by postdoctoral students and/or clinical assistants in psychology - 2 hour group intervention (1 day/week) for 8 weeks   - Sessions focused on development of specific social skills   **Outcomes:**   - Feasibility (satisfaction) - Social skills (SSRS) - HRQoL (PedsQL-Cancer) - Behavior (CBCL, YSR) - Depression (CDI) | **Findings:**   - All participants/parents found intervention helpful for improving interpersonal relationships & helping to express feelings - Minimal attrition with average attendance at 90% and adherence >80% - Significant improvement in social skills (*d*=-.68, *p*=.024) & QoL (*d*=-2.03, *p*=.001) following intervention - No changes in behavior or depression levels - Effects did not persist over time (non-significant at 6-month follow-up)   **Limitations:**   - Lack of control group - Small sample size |
| dos Reis Bigatão (2016)^31^ | 23 participants  Ages 29 to 85 years (mean 55)   - Even gender distribution - Individuals in outpatient setting who were initiating treatment for high-grade glioma   Primary brain tumors  South America (Brazil) | To evaluate the effectiveness of an educational program in terms of fatigue and QoL | **Design:**   - Controlled trial   - 2 study arms (intervention group = education, control group = usual care)   - Timing: during treatment - Informational leaflet provided educating participants about fatigue & participants attended 7 occupational therapy sessions   **Outcomes:**   - Fatigue & QoL (FACIT-F, FACIT-G) - Depression (BDI) | **Findings:**   - No significant improvements in fatigue, QoL, or depression following educational intervention - 56% of participants adhered to the intervention   **Limitations:**   - Small sample size - Limited information about participants - No discussion of confounders |
| Boele (2018)^32^ | 108 participants  Mean age 47 years   - Majority female - Individuals with brain or non-CNS tumors & depressive symptoms   Primary brain tumors & non-CNS tumors  Europe (Netherlands) | To evaluate the effects of an online guided self-help intervention for depressive symptoms in adult glioma patients | **Design:**   - RCT   - 2 study arms (intervention group = gliomas, control group = non-CNS tumors)   - Timing: varied (during and post-treatment) - Internet-based guided self-help intervention done 3-5 times/week for 12 weeks   - Online support from coach (RN or psychology-trained individual)   **Outcomes:**   - Depressive symptoms (CES-D) - Fatigue (CIS) - HRQoL (SF-36) - Feasibility (attrition, adherence) | **Findings:**   - No significant improvements in depressive symptoms or HRQoL for intervention group - Fatigue decreased post-intervention for intervention group (*p*=.05, *d*=.306) - Low adherence to intervention over time (worse in intervention group) & significant attrition   **Limitations:**   - Lack of statistical power - Significant attrition & poor intervention adherence - Continued use of antidepressant medications during intervention may have attenuated findings |
| Richard (2019)^33^ | 25 participants  Ages 21 to 68 years (mean 48)   - Majority male - Individuals in outpatient neuro-oncology clinic with cognitive complaints   Primary brain tumors  North America (Canada) | To evaluate the efficacy of goal management training (GMT) to improve executive dysfunction in brain tumor patients | **Design:**   - Pilot RCT   - 3 study arms (intervention group = GMT, active control group = BHP [no cognitive training], passive control group = usual care)   - Timing: 3+ months after surgery/treatment - 8 weekly 2-hour sessions w/ clinical neuropsychologist (homework in between sessions) for 4 weeks   **Outcomes:**   - Executive functioning (TMT-B, TEA, SART, HT, BADS) - Cognitive symptoms & memory (HVLT-R) - Processing speed (TMT-A) - Emotional functioning & coping/processing (PANAS, HADS, GSES, IIRS) - Feasibility (adherence, retention) | **Findings:**   - Significant improvement in executive functioning for GMT group post-intervention & at follow-up (*p*=.002, *d*=1.09) - All groups had significant improvements in memory - Significant improvement in processing speed for GMT group post-intervention & at follow-up (*p*=.015, *d*=.68), - Only marginal improvement in cognitive symptoms for GMT group post-intervention & at follow-up - Marginal improvements in emotional functioning for GMT group post-intervention & at follow-up, no significant changes in coping/processing for all groups - Adherence & retention high in all groups   **Limitations:**   - Small sample size - Only patients who self-identified cognitive issues were approached |
| Zucchella (2013)^34^ | 53 participants  Mean age 56 years   - Even gender distribution - Individuals in inpatient neurorehabilitation unit following neurosurgery   Primary brain tumors  Europe (Italy) | To test the effectiveness of early cognitive rehabilitation treatment on neuropsychological performance for early post-surgical brain tumor patients | **Design:**   - RCT   - 2 study arms (intervention group = rehabilitation, control group = usual care)   - Timing: within 2 weeks of surgery (no other treatments) - Intervention administered by 2 psychologists   - 16 1-hour sessions 4 days/week for 4 weeks   **Outcomes:**   - Neuropsychological performance (MMSE, digit span & Corsi’s Test, RAVLT, PM47, FAB, TMTA, TMTB, attentive matrices, Rey-Osterrieth complex figure copy, ENPA) | **Findings:**   - Significant improvement in cognitive function for intervention group - Specific cognitive function domains that had significant findings were related to visual attention (TMTA & B, attentive matrices) & verbal memory (RAVLT delayed, logical memory immediate & delayed)   **Limitations:**   - Lack of follow-up to determine intervention durability over time - No QoL or symptom assessments |
| ***PHYSICAL ACTIVITY INTERVENTIONS*** | | |  |  |
| Ayotte (2017)^35^ | 16 participants  Ages 20 to 74 years (mean 54)   - Majority male - Individuals in inpatient rehabilitation facility & followed outpatient   Primary & metastatic brain tumors  North America (United States) | To investigate the feasibility of aerobic exercise training in patients with brain tumors in inpatient rehabilitation & outpatient settings | **Design:**   - Feasibility trial   - Single study arm   - Timing: after surgery (no other treatments) - Training with patients done by 2 physical therapists   - Phase 1=lower extremity ergometry training for 20 min 5 days/week   - Phase 2=outpatient ergometry training 3 days/week   **Outcomes:**   - Feasibility (adherence, retention) - Aerobic capacity (cycle ergometer) & submaximal endurance (6MWT) - Fatigue (FACIT-Fatigue) - Functionality (FIM) - Composite strength of LE muscles (HHD) | **Findings:**   - 100% adherence during phase 1 - Significant improvement in aerobic capacity (*p*=.006) & 6MWT distance (*p*=.006) - Significant improvement in fatigue levels post-training (*p*=.004) - Significant attrition between intervention phases (only 1 participant completed phase 2)   **Limitations:**   - Lack of control group - Small sample size - Unable to assess feasibility of 2 phase intervention due to attrition levels |
| Baima (2017)^36^ | 15 participants  Mean age 55 years   - Even gender distribution - Individuals in outpatient setting who can exercise at home   Primary & metastatic brain tumors  North America (United States) | To evaluate compliance & safety of a home-based exercise program for patients with brain tumors | **Design:**   - Observational trial   - Single study arm   - Timing: w/in 1 month after surgery, during treatment (for 50% of sample) - Patients received 1-time training (watched exercise video and live demo of resistance bands exercises, a balance exercise & walking recommendations)   - Patients instructed to do exercises every day for 1 month   **Outcomes:**   - Feasibility (adherence, retention, interview)) - QoL (FACT-Br) - Self-reported AEs | **Findings:**   - 93% of patients started the exercises during the 1 month study - 64% did exercises ≥ 4 times/week - Higher frequency of exercising associated with social well-being subscale of FACT-Br, being married, and an income > $50K/year   **Limitations:**   - Lack of control group - Small sample size - Did not assess for symptomatic or QoL improvements following intervention |
| Capozzi (2016)^37^ | 24 participants  Mean age 53 years   - Majority male - Individuals in outpatient neuro-oncology clinic who reported fatigue, depression, anxiety, drowsiness, or issues with well-being   Primary brain tumors  North America (Canada) | To assess the feasibility of a clinic-supported 12-week aerobic and resistance exercise program for primary brain tumor patients | **Design:**   - Feasibility trial   - Single study arm   - Timing: during treatment (for 40% of sample) - Aerobic & resistance exercise program (ENHANCE) performed 1 day/week for 12 weeks   - 7-10 min on stationary bike (aerobic), 8-10 min of resistance exercises w/ 3-5 min of moderate activity aerobic exercise for every 3-4 resistance exercises   **Outcomes:**   - Feasibility (recruitment, adherence, patient safety) - Physical fitness (anthropometrics) - Symptoms (ESAS) | **Findings:**   - Average adherence to exercise classes was 61% with 58% attending final session - 1 documented AE (patient fall during exercise session) - Significant improvements reported in grip strength (*p*=.022), performance on sit-to-stand test (*p*=.004), and decreased waist circumference (*p*=.037) - Significant decrease in fatigue (*p*=.002), depression (*p*=.009), drowsiness (*p*=.004), and concern with well-being (*p*=.005)   **Limitations:**   - Lack of control group - Small sample size - Moderate attrition |
| Colledge (2018)^38^ | 48 participants  Mean age 59 years   - Majority female - Individuals in home setting who were willing to work with exercise coach   Post-aneurysm subarachnoid hemorrhage, primary brain tumors, healthy controls  Europe (Switzerland) | To determine if a 12-week personalized exercise intervention has an impact on psychological functioning, verbal learning & memory, subjective sleep & dysfunctional sleep-related cognitions, and objective sleep of patients | **Design:**   - Non-randomized trial   - 3 study arms (intervention group = aSAH, comparator group = meningioma, control group = healthy controls)   - Timing: long-term follow-up after brain surgery - 1 training session per week supervised by trained exercise coach, other 2-4 sessions were unsupervised   - 30-45 min sessions (3-5 days/week) for 12 weeks   **Outcomes:**   - Psychological functioning (BDI, 10-item PSS, Satisfaction with Life Scale) - Verbal memory & learning (VLMT) - Subjective sleep quality (ISI) - Objective sleep quality (SOMNOwatch device) | **Findings:**   - Significant reductions in depressive (*d*=-.352, *d*=-.438) and insomnia symptoms (*d*=-.038, *d*=-.178) for aSAH & meningioma groups, respectively - Perceived stress significantly decreased in meningioma group (*d*=-.073), but increased in aSAH group - Total learning performance significantly increased in all 3 groups - Moderate attrition in both intervention groups   **Limitations:**   - Small sample size - No account for pre-existing psychiatric disease in participants - Problems with missing data |
| Gehring (2018)^39^ | 34 participants  Mean age 49 years   - Majority female - Individuals in outpatient setting who self-reported being inactive or only moderately active & were interested in increasing exercise   Primary brain tumors  Europe (Netherlands) | To investigate the feasibility of a home-based, remotely guided exercise intervention for patients with gliomas | **Design:**   - Pilot RCT   - 2 study arms (intervention group = exercise, active control group = usual lifestyle)   - Timing: on imaging surveillance for 6+ months - Patients wore HR monitors connected to online platform (monitored by physiotherapist) & they kept a log of exercise experiences   - 3 home-based exercise sessions/week at 60-85% max HR for 6 months   **Outcomes:**   - Feasibility (adherence, retention, MET, IPAQ) - Physical fitness (ECG, expert-administered exercise test) - Neuropsychological health (battery) - Satisfaction (questionnaire) | **Findings:**   - Good adherence & retention - After 6 months, cardiorespiratory fitness was significantly higher in exercise group compared to control group (*d*=.24) - Self-reported activity increased in both groups (more so in exercise group) - High patient satisfaction with intervention   **Limitations:**   - Small sample size - Only included patients motivated to exercise - Exercise intervention varied based on patient preferences |
| Gehring (2020)^40^ | 32 participants  Ages 20 to 74 years (mean 54)   - Majority female - Individuals in outpatient setting who self-reported being inactive or only moderately active & were interested in increasing exercise   Primary brain tumors  Europe (Netherlands) | To explore the possible impact of an exercise intervention that is designed to improve cognitive functioning in glioma patients | **Design:**   - Pilot RCT   - 2 study arms (intervention group = exercise, active control group = usual lifestyle)   - Timing: on imaging surveillance for 6+ months - Patients wore HR monitors connected to online platform (monitored by physiotherapist) & kept exercise logs   - 3 home-based sessions/week at 60-85% max HR for 6 months   **Outcomes:**   - Cognitive performance (battery) - Cognitive function (MOS-Cog) - Fatigue (MFI) - Sleep quality (PSQI) - Mood (POMS) - QoL (QLQ-BN, SF-36) | **Findings:**   - Good adherence & retention - Significantly higher cognitive performance in exercise group compared to control group (attention inhibition & span, auditory selective attention & working memory, information processing speed, and executive function) - PROs in more favorable in exercise group (self-reported cognitive function, sleep, fatigue, and mood) - Mood & mental health related QoL better in exercise group compared to control group   **Limitations:**   - Small sample size - Exercise intervention varied based on patient preferences - Effect sizes not provided |
| Hansen (2020)^51^ | 64 participants  Mean age 54 years   - Majority male - Individuals in outpatient rehabilitation facility who were functionally independent   Primary brain tumors  Europe (Denmark) | To assess the effectiveness of a physical therapy- and occupational therapy-based rehabilitation intervention compared with usual rehabilitation care for QoL during active anticancer treatment | **Design:**   - RCT   - 2 study arms (intervention group = rehab intervention, control group = usual care)   - Timing: 1-2 months after surgery (no other treatments) - Training with patients done by trained physical therapists - 90 min exercise intervention (3 days/week) for 6 weeks   - 60 min occupational therapy intervention (2 days/week) for 6 weeks   **Outcomes:**   - QoL (GHS) - HRQoL & symptoms (EORTC BN-20) - Functional performance (ergometer, resistance training test, 10-meter walking test, postural sway) | **Findings:**   - 86% of sample completed intervention & follow-up assessments - Significant improvements in HRQoL (*p*<.01), fatigue (*p*=.04), vision issues (*p*=.02), communication deficits (*p*<.01), headaches (*p*=.03), drowsiness (*p*=.04) & itchy skin (*p*=.02) - Significant improvements in functional performance measures including aerobic power (*p*=.03), elbow extension (*p*=.02) & flexion (*p*<.01), and leg muscle strength (*p*=.02)   **Limitations:**   - Recruitment difficulties - Short intervention period |
| Hojan (2020)^41^ | 54 participants  Mean age 51 years   - Majority male - Individuals in inpatient or outpatient rehabilitation program & in generally good health   Primary brain tumors  Europe (Poland) | To evaluate the effectiveness of a multidisciplinary rehabilitation, carried out as an outpatient or inpatient program, as prevention of disability in brain tumor patients | **Design:**   - Observational trial   - 2 study arms (2 intervention groups [inpatient vs. outpatient rehab])   - Timing: within 1 month after completion of treatment - *Inpatient:*    - Individual 150 min sessions 6 days/week for 12 weeks - *Outpatient:*    - Group 120 min sessions 5 days/week for 12 weeks   **Outcomes:**   - Functionality (FIM, BI, BBS) - Cognitive function (ACE III) - Self-reported functionality (FACT, FACT-Br, FACT-Cog) | **Findings:**   - Significant differences between groups at baseline regarding clinical functional parameters - Significant improvement of motor functioning & in most cognitive functioning domains in both intervention groups - Significant improvement in self-reported functionality in both groups (apart from cognition in outpatient group)   **Limitations:**   - Lack of true control group (no exercise) - No standardization of intervention between groups - Recruitment difficulties |
| Lam (2018)^42^ | 70 participants  Mean age 13 years   - Even gender distribution - Individuals in inpatient oncology ward on active treatment   Primary brain tumors & non-CNS tumors  East Asia (Hong Kong) | To examine the effectiveness of an integrated program in promoting physical activity, reducing fatigue, enhancing physical activity self-efficacy, muscle strength, and quality of life among Chinese children with cancer | **Design:**   - RCT   - 2 study arms (intervention group = exercise, control group = placebo intervention)   - Timing: within 1 month of diagnosis & on active treatment - Integrated program with 28 home visits from coaches over 6 months   - 15-min health education talk   - Deep breathing & relaxation exercises   **Outcomes:**   - Fatigue (BFI) - Physical activity levels (CUHK-PARCY) - Physical activity self-efficacy (PASE) - Muscle strength (dynamometer) - QoL (SF-36) - Feasibility (adherence, AEs) | **Findings:**   - Significant medium-large effects of intervention for cancer-related fatigue (η=.09), physical activity (η=.12) & self-efficacy (η=.11), and bilateral grip strength (.10) & small-medium effect for QoL (η=.05) - Exercise group reported significantly lower levels of fatigue (*p*=.01) & higher levels of self-efficacy (*p*=.003) compared to control group - Bilateral grip strength greater for exercise group compared to controls (*p*=.006) - Exercise group reported better QoL (*p*=.045) compared to control group - High adherence with no AEs reported   **Limitations:**   - Short follow-up period - Levels of physical activity not objectively measured |
| Müller (2016)^43^ | 150 participants  Ages 4 to 18 years (mean 11)   - Majority male - Individuals in inpatient rehabilitation facility who had completed upfront cancer treatments   Primary brain tumors & non-CNS tumors  Europe (Germany) | To evaluate immediate & long-term results of a 4-week inpatient rehabilitation program for children & adolescents after cessation of acute cancer treatment with respect to objectively assessed physical activity | **Design:**   - Observational trial   - 3 study arms (intervention group_1_ = leukemia/lymphoma, intervention group_2_ = brain tumor, intervention group_3_ = sarcoma)   - Timing: long-term follow-up (within 1-2 years of treatment completion) - Individual (physiotherapy) & group activities (exercise training, sports games)   - 2-5 30-60 min sessions/week for 12 weeks   **Outcomes:**   - Physical activity (StepWatch 3 activity monitor) - HRQoL (KINDL) - Feasibility (adherence, retention, questionnaire) | **Findings:**   - Significant effects on physical activity only found at 12-month follow-up for full sample (*p*<.03) - Rehabilitation effects more pronounced for cadence variables in brain tumor & sarcoma patients compared to hematologic cancers - Significant immediate & sustained improvement in HRQoL (*p*<.006) for brain tumor patients   **Limitations:**   - Lack of true control group (no exercise) - Parents completed questionnaires for ages 4-7 - Used 1-min recording intervals for physical activity sensors |
| Nowak (2023)^49^ | 30 participants  Mean age 51 years   - Majority male - Individuals in outpatient exercise clinics who are currently undergoing chemoradiation   Primary brain tumors  Australasia (Australia) | To explore the feasibility & preliminary efficacy of a structured exercise intervention on physical function, body composition, fatigue, sleep quality & QoL in individuals with high-grade glioma undertaking adjuvant chemotherapy | **Design:**   - Feasibility trial   - Single study arm   - Timing: during treatment - Aerobic & resistance training exercises supervised by clinical exercise physiologists   - Training sessions done in small groups (1-4 patients) 2 times/week for 1 hour   **Outcomes:**   - Feasibility (recruitment, retention, safety, adherence & compliance) - Body composition (DEXA) - Fatigue (FACIT-F) - Physical function (400-m walk test, 6MWT, chair rise test, 1-repetition max leg press test) - QoL (SF-36) - Sleep quality (PSQI) | **Findings:**   - 32% of patients withdrew prior to post-intervention assessment timepoint (related to treatment SEs) - On average, participants attended 90% of their training sessions, but only 43% complied with prescribed exercise dosage - No reported AEs related to exercise intervention - Significant improvements reported in leg press strength (*p*=.024) & repeated chair rise time (*p*=.047) - No significant changes in fatigue, sleep quality, QoL, body composition, or other physical function measures   **Limitations:**   - Lack of control group - Small sample size - High attrition rate (though unrelated to intervention) |
| Ovans (2018)^44^ | 15 participants  Ages 7 to 18 years (mean 12)   - Majority male - Individuals in remote (home) environment during or after completion of treatment   Primary brain tumors  North America (United States) | To evaluate a physical activity intervention for children & youth who are currently receiving treatment or have recently completed treatment for a brain tumor | **Design:**   - Pilot trial   - Single study arm   - Timing: within 2 years of diagnosis, on treatment (for 20% of sample) - 12-week intervention using FitBit Flex combined with coaching by physical therapist   - 5 PT coaching sessions over 12-week intervention period   **Outcomes:**   - Feasibility (enrollment, attrition, compliance) - Physical activity (FitBit Flex, GLTEQ) - Functional capacity (6MWT) - QoL (PedsQL Generic Core) - Fatigue (PedsQL Fatigue) | **Findings:**   - 75% of those enrolled completed 12-week intervention & met or exceeded their step goals during 36% of weeks - Significant increase in distance in 6MWT (*p*=.04) - No significant changes in mean # of steps, but higher step counts associated with lower fatigue scores - Significant improvement in total fatigue (*p*=.004), general (*p*=.003), and sleep/rest subscales (*p*=.02), while cognitive fatigue & QoL remained unchanged   **Limitations:**   - Lack of control group - Small sample size - Selection bias |
| Piscione (2017)^45^ | 28 participants  Ages 8 to 17 years (mean 12)   - Majority male - Individuals in outpatient setting in group or group/home setting   Primary brain tumors  North America (Canada) | To examine the efficacy of exercise training for improving physical functioning and cardiopulmonary fitness in survivors of paediatric brain tumors treated with cranial irradiation | **Design:**   - Controlled trial, cross-over design   - 2 study arms (intervention group = immediate exercise training, control group = delayed exercise training)   - Timing: 1+ years since diagnosis & treatment with radiation - Training with patients done by trained physiotherapists in either a group or combined group/home setting   - *Group:* 3 90-min sessions/week for 12 weeks   - *Combined:* 2 90-min group sessions & 2 30-min individual home-based sessions/week   **Outcomes:**   - Feasibility (attrition, adherence) - Physical functioning (BOT-2) | **Findings:**   - Participants completing 84% of all sessions with no attrition - Those in combined setting had significantly higher bilateral coordination scores (*p*=.01) compared to those in group setting - Exercise training resulted in significant improvement in bilateral coordination (*p*=.02) & performance was maintained 12 weeks after training ended - No significant changes in balance, strength, or running speed/agility based on training setting or effects of time   **Limitations:**   - Small sample size - No true control group (non-exercisers) - Assessments not blinded |
| Rath (2018)^46^ | 20 participants  Ages 16 to 24 years (mean 20)   - Even gender distribution - Individuals in outpatient setting who could participate in group exercise program   Primary brain tumors & non-CNS tumors  Australasia (Australia) | To determine feasibility, safety & metabolic as well as psychological impact of a 6-month tailored, supervised, pragmatic exercise program in 15- to 23-year old survivors of childhood cancer | **Design:**   - Feasibility trial   - Single arm design   - Timing: 1+ years since treatment completion - Exercise program consisting of strength-based training & aerobic activity   - 20-30 min intervention (3 days/week) for 24 weeks   **Outcomes:**   - Feasibility (attrition, adherence, AEs) - Metabolic function (stadiometer, weight, waist/hip circumference, OGTT, DEXA, echocardiogram) - Cognitive function (WASI-II) - Adaptive function (ABAS-II) - Mental health (ASEBA) | **Findings:**   - 35% dropped out or did not adhere to intervention - 4 patients required home-based adaptation of intervention - 2 AEs (1 fall related to exercise session, other unrelated to intervention) - Slight improvement in trunk:limb ratio (*p*=.015), but no improvements seen in other body composition measures - Improvement in some aspects of adaptive function, but no significant changes in cognitive function or mental health   **Limitations:**   - Lack of control group - Small sample size - Intervention varied across participants - Time-consuming assessments |
| Sandler (2023)^50^ | 12 participants  Ages 21 to 74 years (mean 51)   - Majority male - Individuals in outpatient clinic-based exercise program   Primary brain tumors  Australasia (Australia) | To determine the safety, feasibility & potential effect of an 18-week exercise intervention for adults with primary brain cancer | **Design:**   - Feasibility trial   - Single study arm   - Timing: within 3-6.5 months after treatment completion - Individually prescribed exercise program developed by an exercise physiologist   - 150 min intervention with 2 resistance training sessions/week for 18 weeks   **Outcomes:**   - Feasibility (retention, adherence, compliance, AEs) - QoL (FACT-Br) - Fatigue (FACIT-F) - Depression & anxiety (HADS) - BMI (height, weight) - Aerobic fitness (6MWT) - Physical function (single leg balance, 12-item performance battery) - Muscular strength (hand-grip strength, row & leg press) | **Findings:**   - 83% of participants achieved average of ≥ 150-min of exercise/week - 9 exercise-related AEs reported by 5 participants (all non-serious, grades 1-2) - Significant improvements seen post-intervention in QoL (*p*=.01), depression (*p*=.03) & weekly physical activity (*p*=.002) with sustained effects at 6 months - Significant improvements in aerobic fitness (*p*=.002), physical function (*p*=.002) & physical performance (*p*<.001)   **Limitations:**   - Lack of control group - Small sample size |
| Spencer (2021)^47^ | 17 participants  Mean age 59 years   - Majority male - Individuals receiving treatment for a brain tumor in outpatient setting   Primary brain tumors  North America (United States) | To assess the feasibility to recruit & retain participants with high-grade glioma into a 10-week exercise intervention & evaluate effects on cancer-related fatigue & QoL | **Design:**   - Pilot controlled trial   - 3 study arms (intervention group_1_ = exercise, intervention group_2_ = education, control group = usual care)   - Timing: during treatment - Education group completed 60-min session & kept exercise log - Exercise group completed 150 min of exercise & 2 strength training sessions/week, info on setting up FitBit Alta, and kept exercise log   **Outcomes:**   - Feasibility (accrual, retention) - Engagement (weekly logs) - QoL (EORTC QLQ-C30) - Fatigue (VAFS) - Cardiorespiratory fitness & strength (6MWT, sit & reach test, hand grip test, push-up test, FitBit Alta HR tracker) | **Findings:**   - Participants attended 80% of in-person exercise classes & 90% completed weekly logs - No exercise-related AEs reported - Significant improvements in fatigue, QoL, cardiorespiratory fitness, flexibility & strength in exercise group compared to other 2 groups   **Limitations:**   - Small sample size - Short intervention period with no long-term follow-up - Significance threshold not provided for statistical tests |
| Troschel (2020)^48^ | 15 participants  Ages 29 to 77 years (mean 48)   - Even gender distribution - Individuals in outpatient setting willing to participate in 1-week ski intervention   Primary brain tumors & participant relatives  Europe (Germany) | To describe the feasibility & safety of a ski-based exercise intervention in brain tumor patients and their relatives | **Design:**   - Pilot trial   - Single study arm   - Timing: during treatment (for 50% of patients) - Training with patients/relatives done by ski instructors for 2 hours twice/daily for 1 week under supervision of physicians   - Participants wore a Polar Electro fitness watch during the day to track activity levels   **Outcomes:**   - Feasibility (adherence, retention, AEs) - Physical exercise (Polar Electro watch) - QoL (EORTC QLQ-C30) - Well-being (WHO-5) - Distress (DT) - Self-efficacy (ASKU) - Anxiety & depression (HADS) | **Findings:**   - No severe AEs reported related to intervention - Physical exercise increased during intervention & remained elevated 1 week afterwards in patient group, no change for relatives - QoL, well-being, distress, anxiety & depression all significantly improved during the intervention - Relatives reported worse anxiety, depression & distress compared to patients   **Limitations:**   - Lack of control group - Small sample size - No robust statistical analyses performed - Lack of control for confounders |

*Abbreviations:* CNS: central nervous system; RCT: randomized controlled trial; RN: registered nurse; CES-D: Center for Epidemiological Studies-Depression, CIS: Checklist for Individual Strength-Fatigue; HRQoL: health-related quality of life; SF-36: 36-item Short-Form Survey; ABT: app-based training; HADS: Hospital Anxiety and Depression Scale; FACT-Br: Functional Assessment of Cancer Therapy-Brain; SF-12: 12-item Short Form Survey; FFMQ: Five Facet Mindfulness Questionnaire; GMT: goal management training; BHP: Brain Health Program; TMT: Trail Making Test, TEA: Test of Everyday Attention; SART: Sustained Attention to Response Task; HT: Hotel Test; BADS: Behavioral Assessment of the Dysexecutive Syndrome; HLVT: Hopkins Verbal Learning Test; PANAS: Positive and Negative Affect Schedule; GSES: General Self-Efficacy Scale; IIRS: Illness Intrusiveness Ratings Scale; MMSE: Mini Mental State Exam; RAVLT: Rey Auditory Verbal Learning Test; PM47: Progressive Matrices 47; FAB: Frontal Assessment Battery; ENPA: Esame Neuropsicologico per l’Afasia; QoL: quality of life; 6MWT: 6-minute walk test; FACIT-Fatigue: Functional Assessment of Chronic Illness Therapy-Fatigue; FIM: Functional Independence Measure; LE: lower extremity; HHD: hand-held dynamometer; AEs: adverse events; ESAS: Edmonton Symptom Assessment System; aSAH: aneurysmal subarachnoid hemmorhage; BDI: Beck Depression Inventory; PSS: Perceived Stress Scale; VLMT: Verbaler Lern-und Merkfahigkeitstest (translation of AVLT); ISI: insomnia severity index; HR: heart rate; MET: metabolic equivalent of task; IPAQ: International Physical Activity Questionnaire; ECG: electrocardiogram; MOS-Cog: Medical Outcomes Study-Cognitive Functioning Scale; MFI: Multidimensional Fatigue Inventory; PSQI: Pittsburgh Sleep Quality Index; POMS: Profile of Mood States; QLQ-BN: Quality of Life in Brain Cancer; BI: Barthel Index; BBS: Berg Balance Scale; ACE III: Addenbrooke’s Cognitive Examination III; BFI: Brief Fatigue Inventory; CUHK-PARCY: Chinese University of Hong Kong Physical Activity Rating for Children and Youth; PASE: Physical Activity Self-Efficacy scale; KINDL: German instrument to measure HRQoL in children; DEXA: dual-energy X-ray absorptiometry; GLTEQ: Godin-Shephard Leisure Time Exercise Questionnaire; PedsQL: Pediatric Quality of Life Inventory; BOT-2: Bruininks-Oseretsky Test of Motor Proficiency 2^nd^ edition; OGTT: oral glucose tolerance test; WASI-II: Wechsler Abbreviated Scale of Intelligence 2^nd^ edition; ABAS-II: Adaptive Behavior Assessment System 2^nd^ edition; ASEBA: Achenbach System of Empirically Based Assessment; BMI: body mass index; EORTC QLQ-C30: European Organisation for Research and Treatment of Cancer Quality of Life Questionnaire; VAFS: visual analog fatigue scale; WHO-5: World Health Organization Five Well Being Index; DT: Distress Thermometer; ASKU: Allgemeine-Selbstwirksamkeit-Kurzskala scale; GHS: global health status; SSRS: Social Skills Rating Scale; CBCL: Child Behavior Check List; YSR: Youth Self-Report instrument; CDI: Children’s Depression Inventory; RSES: Rosenberg Self-Esteem Scale; MDASI: MD Anderson Symptom Inventory; VAS: Visual Analogue Scale; LOS: length of stay
