# Supplementary material for: Landscape and impact of mind-body, cognitive-behavioral, and physical activity interventions in adolescent and adult brain tumor patients: A systematic review
Source: Neurooncol Adv. 2024 Aug 22;6(1):vdae134. doi: 10.1093/noajnl/vdae134 (PMC11445902; doi:10.1093/noajnl/vdae134)
Supplement: vdae134_suppl_Supplementary_Table3 [file vdae134_suppl_supplementary_table3.docx]

**Supplementary Table 3. Risk of bias appraisal for included literature using ROBINS-I tool**

| **Included Literature** (N=29) | **Risk of Bias Domains** | **Domain Appraisal** | **Domain Bias Judgment** | **Overall Bias Judgment** | **Support for Judgment** |
| --- | --- | --- | --- | --- | --- |
| *Barrera (2009)^30^* | ***Pre-Intervention*** |  |  | **MODERATE** |  |
|  | Bias due to confounding | PY | Moderate |  | only controlled for passage of time and maturation, not all confounding domains |
| Non-randomized trial | Bias in selection of participants | PY | Low |  | possible selection bias due to recruitment approach |
|  | ***At intervention*** |  |  |  |  |
|  | Bias in classification of interventions | N | Low |  | only 1 intervention, and it was clearly defined |
|  | ***Post-intervention*** |  |  |  |  |
|  | Bias due to deviations from intended interventions | PN | Low |  | only 1 intervention, intervention was successfully implemented for most participants (there was imperfect compliance) |
|  | Bias due to missing data | PN | Low |  | 17/19 participants (89%) were contacted for follow-up, only 1 survivor dropped |
|  | Bias in measurement of outcomes | PN | Low |  | negligible assessor judgment in outcome measures, research assistant blind to details of group intervention & study purpose coded logs for adherence |
|  | Bias in selection of reported results | PY | Moderate |  | only following up on the participants that showed significant effects could have introduced bias |
| *Colledge (2018)^38^* | ***Pre-Intervention*** |  |  | **MODERATE** |  |
|  | Bias due to confounding | Y | Moderate |  | potential for confounding bias & no statistical correction for multiple tests run |
| Non-randomized trial | Bias in selection of participants | Y | Moderate |  | authors acknowledged a degree of selection bias |
|  | ***At intervention*** |  |  |  |  |
|  | Bias in classification of interventions | PN | Low |  | intervention was clearly defined, but some of the training sessions were supervised and some were not, so there were differences in the intervention across groups |
|  | ***Post-intervention*** |  |  |  |  |
|  | Bias due to deviations from intended interventions | PN | Low |  | only 1 intervention but the research assistants were not blinded to participants' diagnosis |
|  | Bias due to missing data | PY | Low |  | 72% of participants took part in the post-test assessments + 5% more lost to 6 month follow up, authors acknowledge potential bias of missing data |
|  | Bias in measurement of outcomes | PN | Low |  | research assistants were not blinded to participants' diagnosis but the outcomes were not subjective and they did not use different methods to assess outcomes |
|  | Bias in selection of reported results | PN | Low |  | authors reported on outcomes for participants that dropped out, which reduces potential for bias in selection of reported results |
| *Hojan (2020)^41^* | ***Pre-Intervention*** |  |  | **MODERATE** |  |
|  | Bias due to confounding | PY | Moderate |  | potential for confounding bias due to uncontrolled for factors |
| Non-randomized trial | Bias in selection of participants | PY | Moderate |  | numerous participants dropped out throughout the course of rehab and the authors excluded their data from the analysis |
|  | ***At intervention*** |  |  |  |  |
|  | Bias in classification of interventions | PY | Moderate |  | no control group (those who didn't participate in any rehab), the intervention groups were well defined |
|  | ***Post-intervention*** |  |  |  |  |
|  | Bias due to deviations from intended interventions | PY | Moderate |  | many participants dropped out in both groups during the rehab intervention |
|  | Bias due to missing data | Y | Serious |  | nearly 50% total attrition across groups, which could significantly bias the results due to this missing data |
|  | Bias in measurement of outcomes | PN | Low |  | outcomes measurements were consistent across the groups, the assessors were aware of the interventions received by the participants |
|  | Bias in selection of reported results | PY | Moderate |  | no corrections done for multiple comparisons |
| *Baima (2017)^36^* | ***Pre-Intervention*** |  |  | **SERIOUS** |  |
|  | Bias due to confounding | PY | Low |  | some adjustment for potential confounders, no accounts for the effects of time |
| Non-randomized trial | Bias in selection of participants | PY | Moderate |  | providers were able to decline approaching participants (based on health status, participants who couldn't perform home exercises were excluded |
|  | ***At intervention*** |  |  |  |  |
|  | Bias in classification of interventions | PY | Moderate |  | no control group, intervention groups were clearly defined |
|  | ***Post-intervention*** |  |  |  |  |
|  | Bias due to deviations from intended interventions | PY | Low |  | 2/3 of participants completed intervention period and continued to exercise regularly |
|  | Bias due to missing data | Y | Serious |  | 1/3 of participants did not complete follow-up assessments, analysis not robust to degree of missing data |
|  | Bias in measurement of outcomes | PY | Low |  | outcomes measurement consistent across groups, assessors were aware of intervention received by participants, outcome assessment scores not reported (only *p*-values) |
|  | Bias in selection of reported results | Y | Serious |  | multivariate analysis not performed, outcomes data only reported from small subgroup who completed intervention & continued to exercise |
| *dos Reis Bigatao (2016)^31^* | ***Pre-Intervention*** |  |  | **MODERATE** |  |
|  | Bias due to confounding | PY | Moderate |  | participants randomly assigned to 1 of 2 groups |
| Randomized (but not controlled) trial | Bias in selection of participants | PY | Moderate |  | selection of participants was related to both intervention and outcome |
|  | ***At intervention*** |  |  |  |  |
|  | Bias in classification of interventions | PY | Moderate |  | participants randomly assigned, intervention status clearly defined, but no control group |
|  | ***Post-intervention*** |  |  |  |  |
|  | Bias due to deviations from intended interventions | PY | Moderate |  | only 1 intervention, clearly defined |
|  | Bias due to missing data | PY | Moderate |  | 50% of participants in each group died or dropped out due to clinical reasons |
|  | Bias in measurement of outcomes | PY | Moderate |  | outcomes measurements consistent across the groups, the assessors were aware of the intervention (read them to patient) |
|  | Bias in selection of reported results | PN | Low |  | mixed-effects regression model used for comparisons b/w groups and time points, reported all results including non-significant ones |
| *Govardhan (2019)^25^* | ***Pre-Intervention*** |  |  | **MODERATE** |  |
|  | Bias due to confounding | PY | Moderate |  | no account for the effects of time, no adjustment for potential confounders |
| Non-randomized trial | Bias in selection of participants | PY | Moderate |  | patients screened from inpatient department, could have contribute to selection bias |
|  | ***At intervention*** |  |  |  |  |
|  | Bias in classification of interventions | PY | Moderate |  | no control group, only 1 intervention, intervention was clearly defined |
|  | ***Post-intervention*** |  |  |  |  |
|  | Bias due to deviations from intended interventions | PN | Low |  | all patients completed minimal required attendance of intervention |
|  | Bias due to missing data | PY | Moderate |  | lots of baseline characteristics were excluded, no standard questionnaires |
|  | Bias in measurement of outcomes | PY | Moderate |  | assessors aware of intervention received. |
|  | Bias in selection of reported results | PY | Moderate |  | no robust analysis performed, but raw data and *t*-values provided |
| *Ayotte (2017)^35^* | ***Pre-Intervention*** |  |  | **SERIOUS** |  |
|  | Bias due to confounding | Y | Serious |  | didn't measure pre-intervention variables, patients were also concurrently participating in other therapies (likely many confounders for results) |
| Non-randomized trial | Bias in selection of participants | PY | Moderate |  | very rigid eligibility criteria, may cause significant selection bias |
|  | ***At intervention*** |  |  |  |  |
|  | Bias in classification of interventions | PY | Moderate |  | only 1 intervention, clearly defined |
|  | ***Post-intervention*** |  |  |  |  |
|  | Bias due to deviations from intended interventions | Y | Serious |  | only 1 out of 16 participants completed phase 2 (the outpatient intervention setting) |
|  | Bias due to missing data | PY | Moderate |  | 20 participants enrolled, 4 dropped out during intervention so their data was excluded |
|  | Bias in measurement of outcomes | PY | Moderate |  | outcome assessors aware of intervention |
|  | Bias in selection of reported results | PN | Low |  | results reported appropriately & made available |
| *Capozzi (2016)^37^* | ***Pre-Intervention*** |  |  | **MODERATE** |  |
|  | Bias due to confounding | PY | Moderate |  | no adjustment for potential confounders |
| Non-randomized trial | Bias in selection of participants | PY | Moderate |  | selection based on clinician referral, but inclusion criteria pretty broad |
|  | ***At intervention*** |  |  |  |  |
|  | Bias in classification of interventions | PN | Moderate |  | no control or comparison group, exercise intervention clearly defined |
|  | ***Post-intervention*** |  |  |  |  |
|  | Bias due to deviations from intended interventions | PY | Moderate |  | 1 adverse effect (patient fell), 61% adhered to class, 58% attended final assessment |
|  | Bias due to missing data | PY | Moderate |  |  |
|  | Bias in measurement of outcomes | PY | Moderate |  | outcomes measurement consistent |
|  | Bias in selection of reported results | PY | Moderate |  | didn't include fitness data on patients who didn't adhere to program/attended final assessment |
| *De Tomassi (2020)^24^* | ***Pre-Intervention*** |  |  | **SERIOUS** |  |
| Non-randomized trial | Bias due to confounding | Y | Serious |  | recruited patients w/in average 4.5 days after cranial surgery (likely many confounding variables related to surgery & subsequent treatment), no adjustment for the potential confounders, no adjustment for time |
|  | Bias in selection of participants | Y | Serious |  | for most participants who didn't complete treatment it was b/c of fatigue secondary to tumor or its treatment and recruiting them soon after cranial surgery, selection of participants related to both intervention and outcome, a potentially important amount of follow-up time is missing from analyses |
|  | ***At intervention*** |  |  |  |  |
|  | Bias in classification of interventions | PY | Low |  | no control group, intervention well-defined |
|  | ***Post-intervention*** |  |  |  |  |
|  | Bias due to deviations from intended interventions | Y | Serious |  | significant retention issues |
|  | Bias due to missing data | Y | Serious |  | pre-treatment questionnaire booklets missing for 2 participants, 3 participants died during the 8 week treatment |
|  | Bias in measurement of outcomes | PY | Moderate |  | outcome assessors were aware of the intervention status |
|  | Bias in selection of reported results | PN | Low |  | reported results they had appropriately |
| *Spencer (2021)^47^* | ***Pre-Intervention*** |  |  | **MODERATE** |  |
|  | Bias due to confounding | PY | Moderate |  | did not control for all important confounding domains |
| Non-randomized trial | Bias in selection of participants | PN | Low |  | participants recruited through fliers in cancer center, potential for selection bias |
|  | ***At intervention*** |  |  |  |  |
|  | Bias in classification of interventions | PN | Low |  | control and comparison groups, intervention clearly defined |
|  | ***Post-intervention*** |  |  |  |  |
|  | Bias due to deviations from intended interventions | PY | Moderate |  | COVID restrictions limited some participants from participation |
|  | Bias due to missing data | PN | Low |  | data reasonably complete |
|  | Bias in measurement of outcomes | PY | Moderate |  | control group was not given the same measures assessing cardiorespiratory fitness, flexibility, and strength, all measures administered by PI though, assessors aware of status |
|  | Bias in selection of reported results | PN | Low |  | reported results correspond to all intended outcomes, analyses, and subcohorts |
| *Piscione (2017)^45^* | ***Pre-Intervention*** |  |  | **MODERATE** |  |
| (trial randomization not known) | Bias due to confounding | PY | Moderate |  | controlled for time, baseline performance, gender, handedness, BMI, age at baseline, age at diagnosis, and time since diagnosis |
|  | Bias in selection of participants | PY | Moderate |  | participants may have been predisposed and/or more motivated to want to improve fitness and physical functioning |
|  | ***At intervention*** |  |  |  |  |
|  | Bias in classification of interventions | PY | Moderate |  | intervention groups are poorly defined, they don't state the purpose of the different intervention groups, nor do they state how participants were assigned to either group (randomization not known), consort diagram doesn't clearly define the interventions |
|  | ***Post-intervention*** |  |  |  |  |
|  | Bias due to deviations from intended interventions | PY | Moderate |  | difficult to assess given that interventions were poorly described |
|  | Bias due to missing data | PN | Low |  | adherence to training was 84% |
|  | Bias in measurement of outcomes | PY | Moderate |  | assessors were not blinded, potential performance bias |
|  | Bias in selection of reported results | PN | Low |  | reported results correspond to all intended outcomes, analyses, and subcohorts |
| *Troschel (2020)^48^* | ***Pre-Intervention*** |  |  | **MODERATE** |  |
|  | Bias due to confounding | PY | Moderate |  | did not control for all important confounding domains |
| Non-randomized trial | Bias in selection of participants | PY | Moderate |  | participants recruited from psycho-oncology email list, likely selection bias |
|  | ***At intervention*** |  |  |  |  |
|  | Bias in classification of interventions | PY | Moderate |  | no control group, intervention clearly defined |
|  | ***Post-intervention*** |  |  |  |  |
|  | Bias due to deviations from intended interventions | PY | Moderate |  | possible unplanned co-interventions, like social interaction, departure from everyday life |
|  | Bias due to missing data | PY | Moderate |  | 1 patient passed away before intervention, 1 did not wear watch for at least 60 hours over each 4 day interval |
|  | Bias in measurement of outcomes | PY | Moderate |  | both patients and relatives received same outcome questionnaires but outcome measures could have been influenced by knowledge of intended intervention effect |
|  | Bias in selection of reported results | PY | Moderate |  | no detailed statistical analysis |
| *Rath (2018)^46^* | ***Pre-Intervention*** |  |  | **MODERATE** |  |
| Non-randomized trial | Bias due to confounding | PY | Moderate |  | evidence of time-varying confounding where the intervention received changed over time, 2 participants were offered home-based programs after they said they were unable to regularly attend the group sessions |
|  | Bias in selection of participants | PY | Moderate |  | used an oncology database of the only pediatric institution servicing Western Australia, likely selection bias |
|  | ***At intervention*** |  |  |  |  |
|  | Bias in classification of interventions | PY | Moderate |  | some aspects of the assignments of intervention status were determined retrospectively (see above) |
|  | ***Post-intervention*** |  |  |  |  |
|  | Bias due to deviations from intended interventions | PY | Moderate |  | healthcare providers were not blinded, only 65% of participants were adherent to the program |
|  | Bias due to missing data | PY | Moderate |  | significant missing data due to poor intervention compliance |
|  | Bias in measurement of outcomes | PY | Moderate |  | outcomes likely biased due to varying interventions being administered |
|  | Bias in selection of reported results | PN | Low |  | reported results correspond to all intended outcomes, analyses, and subcohorts |
| *Muller (2016)^43^* | ***Pre-Intervention*** |  |  | **SERIOUS** |  |
|  | Bias due to confounding | PN | Low |  | robust control for confounding variables |
| Non-randomized trial | Bias in selection of participants | PY | Moderate |  | all participants who would've been eligible were included in study |
|  | ***At intervention*** |  |  |  |  |
|  | Bias in classification of interventions | PN | Low |  | only 1 intervention that was clearly defined |
|  | ***Post-intervention*** |  |  |  |  |
|  | Bias due to deviations from intended interventions | PY | Moderate |  | 90 participants, who were enrolled at baseline, were not included in final analysis//did not complete |
|  | Bias due to missing data | Y | Serious |  | 90 participants, who were enrolled at baseline, were not included in final analysis//did not complete yielding significant missing data & source for bias |
|  | Bias in measurement of outcomes | Y | Serious |  | see above |
|  | Bias in selection of reported results | PN | Low |  | robust statistical analyses used for data that was available |
| *Ovans (2018)^44^* | ***Pre-Intervention*** |  |  | **MODERATE** |  |
|  | Bias due to confounding | PY | Moderate |  | did not control for all potential confounding domains, like what the participants' level of PA was pre-diagnosis |
| Non-randomized trial | Bias in selection of participants | PY | Moderate |  | authors acknowledged that patients who chose to participate were likely physically and psychosocially ready to participate in this kind of intervention |
|  | ***At intervention*** |  |  |  |  |
|  | Bias in classification of interventions | PN | Low |  | Intervention group clearly defined, info used to define intervention groups recorded at start |
|  | ***Post-intervention*** |  |  |  |  |
|  | Bias due to deviations from intended interventions | PY | Moderate |  | only 55% of participants adhered to program |
|  | Bias due to missing data | PY | Moderate |  | significant missing data due to poor intervention adherence by participants |
|  | Bias in measurement of outcomes | PY | Moderate |  | outcome assessors are aware of intervention status |
|  | Bias in selection of reported results | PY | Moderate |  | authors did not report results for those who only completed 12 weeks of study participation, omission may introduce bias in findings |
| *Keir (2012)^26^* | ***Pre-Intervention*** |  |  | **MODERATE** |  |
| Non-randomized trial | Bias due to confounding | PY | Moderate |  | consideration for confounders that could contribute to distress, did not control for time effects |
|  | Bias in selection of participants | PY | Moderate |  | participant recruitment limited to 60 mile radius around treatment facility, may have led to selection bias |
|  | ***At intervention*** |  |  |  |  |
|  | Bias in classification of interventions | PN | Low |  | intervention group clearly defined |
|  | ***Post-intervention*** |  |  |  |  |
|  | Bias due to deviations from intended interventions | PN | Low |  | 25 out of 26 eligible patients adhered to intervention schedule |
|  | Bias due to missing data | PN | Low |  | no substantial missing data |
|  | Bias in measurement of outcomes | PY | Moderate |  | outcome measures were all self-reported by the participants, responses could have been influenced by knowledge of the intervention received |
|  | Bias in selection of reported results | PN | Low |  | reported results correspond to all intended outcomes, analyses, and subcohorts |
| *Milbury (2018)^27^* | ***Pre-Intervention*** |  |  | **SERIOUS** |  |
|  | Bias due to confounding | PY | Moderate |  | did not control for time effects (patients may improve as a function of time) |
| Non-randomized trial | Bias in selection of participants | PY | Moderate |  | participants may have been those who were super willing and ready to do yoga, KPS inclusion criteria was super high so may have excluded other HGG pts |
|  | ***At intervention*** |  |  |  |  |
|  | Bias in classification of interventions | PN | Low |  | intervention group clearly defined |
|  | ***Post-intervention*** |  |  |  |  |
|  | Bias due to deviations from intended interventions | PN | Low |  | no significant deviations from intended intervention |
|  | Bias due to missing data | PN | Low |  | outcomes data available for nearly all participants |
|  | Bias in measurement of outcomes | PY | Serious |  | outcome assessors (participants) aware of the intervention received by study participants (themselves) since they reported the outcomes via questionnaire |
|  | Bias in selection of reported results | PN | Low` |  | reported results correspond to all intended outcomes, analyses, and subcohorts |
| *Nowak (2023)^49^* | ***Pre-Intervention*** |  |  | **SERIOUS** |  |
|  | Bias due to confounding | PY | Moderate |  | did not control for all important confounding domains |
| Non-randomized trial | Bias in selection of participants | Y | Serious |  | possible selection bias due to recruitment approach |
|  | ***At intervention*** |  |  |  |  |
|  | Bias in classification of interventions | PN | Low |  | intervention groups clearly defined |
|  | ***Post-intervention*** |  |  |  |  |
|  | Bias due to deviations from intended interventions | Y | Serious |  | only about ½ of enrolled patients participated in exercise program |
|  | Bias due to missing data | Y | Serious |  | many participants dropped out throughout the course of rehab and the authors excluded their data from the analysis, significant missing data as a result |
|  | Bias in measurement of outcomes | PY | Low |  | outcomes measurement consistent across groups, assessors were aware of intervention received by participants |
|  | Bias in selection of reported results | PN | Low |  | reported results correspond to all intended outcomes and analyses |
| *Sandler (2023)^50^* | ***Pre-Intervention*** |  |  | **MODERATE** |  |
|  | Bias due to confounding | PY | Moderate |  | adjusted for some confounding factors (time since dx and treatment) but did not control for all important confounding domains |
| Non-randomized trial | Bias in selection of participants | PY | Moderate |  | patients were recruited at the discretion of nurses/ practitioners at routine appointments, therefore not all eligible patients could have participated (based on whether or not they had an appointment) |
|  | ***At intervention*** |  |  |  |  |
|  | Bias in classification of interventions | PN | Low |  | intervention groups clearly defined |
|  | ***Post-intervention*** |  |  |  |  |
|  | Bias due to deviations from intended interventions | PY | Moderate |  | 58% of patients reported a total of 27 adverse effects , 2 patients withdrew from study |
|  | Bias due to missing data | PN | Low |  | adherence to intervention was 83% |
|  | Bias in measurement of outcomes | PY | Moderate |  | participants received slightly different interventions (PA program was individualized for each patient) |
|  | Bias in selection of reported results | PN | Low |  | reported results correspond to all intended outcomes, analyses, and subcohorts |
| *Gehring (2020)^40^* | ***Pre-Intervention*** |  |  | **MODERATE** |  |
| RCT | Bias due to confounding | PN | Low |  | robust analysis methods to control for potential confounders |
|  | Bias in selection of participants | PY | Moderate |  | recruitment phase may have been perceived as too intimidating for less motivated patients |
|  | ***At intervention*** |  |  |  |  |
|  | Bias in classification of interventions | PN | Low |  | interventions actively assigned by researcher & recorded accurately |
|  | ***Post-intervention*** |  |  |  |  |
|  | Bias due to deviations from intended interventions | PN | Low |  | no deviations from intended intervention, majority of study participants adhered to assigned intervention regime |
|  | Bias due to missing data | PN | Low |  | outcome data available for all or nearly all participants |
|  | Bias in measurement of outcomes | PY | Moderate |  | neither patients nor assessors were blinded to group allocation (except for sports doctor performing the CPETs) |
|  | Bias in selection of reported results | PN | Low |  | reported results correspond to all intended outcomes, analyses, and subcohorts |
| *Lam (2018)^42^* | ***Pre-Intervention*** |  |  | **MODERATE** |  |
| RCT | Bias due to confounding | PN | Low |  | robust analysis methods to control for potential confounders |
|  | Bias in selection of participants | PY | Moderate |  | all participants who would've been eligible for target trial were included in the study |
|  | ***At intervention*** |  |  |  |  |
|  | Bias in classification of interventions | PN | Low |  | intervention status well-defined & intervention definition based solely on info collected at time of intervention |
|  | ***Post-intervention*** |  |  |  |  |
|  | Bias due to deviations from intended interventions | PY | Moderate |  | participants might have revealed the intervention they received to outcome assessors during data collection, so blinding might not have been fully maintained throughout the study |
|  | Bias due to missing data | PN | Low |  | outcome data available for all or nearly all participants |
|  | Bias in measurement of outcomes | PY | Moderate |  | levels of physical activity weren't objectively measured (they were self-reported) |
|  | Bias in selection of reported results | PN | Low |  | reported results correspond to all intended outcomes, analyses, and subcohorts |
| *Zucchella (2013)^34^* | ***Pre-Intervention*** |  |  | **MODERATE** |  |
| RCT | Bias due to confounding | PN | Low |  | robust analysis methods to control for potential confounders |
|  | Bias in selection of participants | PN | Low |  | all participants who would've been eligible for target trial were included in the study |
|  | ***At intervention*** |  |  |  |  |
|  | Bias in classification of interventions | PN | Low |  | intervention status well defined & intervention definition based solely on info collected at time of intervention |
|  | ***Post-intervention*** |  |  |  |  |
|  | Bias due to deviations from intended interventions | PN | Low |  | no deviations from intended, intervention implemented successfully for most participants (83% in study group and 88% in control group) |
|  | Bias due to missing data | PY | Moderate |  | outcome data available for all or nearly all participants |
|  | Bias in measurement of outcomes | PY | Moderate |  | authors acknowledge that some patients may not have been able to express their opinions fully due to cognitive deficits, which may have impacted outcomes of study |
|  | Bias in selection of reported results | PN | Low |  | reported results correspond to all intended outcomes, analyses, and subcohorts |
| *Richard (2019)^33^* | ***Pre-Intervention*** |  |  | **MODERATE** |  |
| RCT | Bias due to confounding | PN | Low |  | robust analysis methods to control for potential confounders |
|  | Bias in selection of participants | PY | Moderate |  | participation was restricted to patients self-identifying w/ cognitive concerns to ensure sufficient insight & motivation to engage w/ interventions, resulting in limited sample size & generalizability to pts lacking insight |
|  | ***At intervention*** |  |  |  |  |
|  | Bias in classification of interventions | PN | Low |  | intervention status well defined & intervention definition based solely on info collected at time of intervention |
|  | ***Post-intervention*** |  |  |  |  |
|  | Bias due to deviations from intended interventions | PN | Low |  | no deviations from intended intervention, a research assistant blind to the participant's group conducted the assessments |
|  | Bias due to missing data | PN | Low |  | outcome data available for all or nearly all participants |
|  | Bias in measurement of outcomes | PY | Moderate |  | some of the outcome measures were PROs (subject to recall bias) |
|  | Bias in selection of reported results | PN | Low |  | reported results correspond to all intended outcomes, analyses, and subcohorts |
| *Hansen (2020)^51^* | ***Pre-Intervention*** |  |  | **MODERATE** |  |
| RCT | Bias due to confounding | PN | Low |  | robust analysis methods to control for potential confounders |
|  | Bias in selection of participants | PY | Moderate |  | authors acknowledged that study population was quite homogenous, which could reduce generalizability |
|  | ***At intervention*** |  |  |  |  |
|  | Bias in classification of interventions | PN | Low |  | intervention status well defined & intervention definition based solely on info collected at time of intervention |
|  | ***Post-intervention*** |  |  |  |  |
|  | Bias due to deviations from intended interventions | PY | Moderate |  | final sample size included just 38% of the required sample size, and only 24% of people screened for eligibility were included |
|  | Bias due to missing data | PY | Moderate |  | due to poor enrollment, lots of missing data for planned analyses |
|  | Bias in measurement of outcomes | PY | Moderate |  | outcome measures were self-reported (subject to recall bias) |
|  | Bias in selection of reported results | PN | Low |  | reported results correspond to all intended outcomes, analyses, and subcohorts |
| *Walworth (2008)^29^* | ***Pre-Intervention*** |  |  | **SERIOUS** |  |
| RCT | Bias due to confounding | PY | Serious |  | did not control for all important confounding domains (such as type of surgery they had) |
|  | Bias in selection of participants | PY | Serious |  | do not describe how they approached the patients, eligibility criteria, or any demographic information of the sample |
|  | ***At intervention*** |  |  |  |  |
|  | Bias in classification of interventions | PN | Low |  | intervention status well defined |
|  | ***Post-intervention*** |  |  |  |  |
|  | Bias due to deviations from intended interventions | Y | Serious |  | music sessions were interrupted, 1 patient removed himself as a participant b/c he was a control and wanted to receive music therapy, assessors not blinded |
|  | Bias due to missing data | PY | Moderate |  | incomplete data collection (as described above) |
|  | Bias in measurement of outcomes | PY | Moderate |  | outcome assessors aware of intervention status, outcome measures were self-reported (subject to recall bias) |
|  | Bias in selection of reported results | PY | Moderate |  | there is no table for the control group's QoL scores |
| *Cheung (2019)^23^* | ***Pre-Intervention*** |  |  | **MODERATE** |  |
| RCT | Bias due to confounding | PN | Low |  | robust analysis methods to control for potential confounders |
|  | Bias in selection of participants | PY | Moderate |  | excluded those w/ musical training, so not all participants who were eligible for the trial were included in the intervention |
|  | ***At intervention*** |  |  |  |  |
|  | Bias in classification of interventions | PN | Low |  | intervention groups clearly defined |
|  | ***Post-intervention*** |  |  |  |  |
|  | Bias due to deviations from intended interventions | PN | Low |  | no deviations from intended intervention, intervention implemented successfully for most participants |
|  | Bias due to missing data | PN | Low |  | participants missed some sessions, which led to some missing data & risk of biasing results |
|  | Bias in measurement of outcomes | PY | Moderate |  | outcome measures were self-reported (subject to recall bias) |
|  | Bias in selection of reported results | PN | Low |  | reported results correspond to all intended outcomes, analyses, and subcohorts |
| *Gehring (2018)^39^* | ***Pre-Intervention*** |  |  | **MODERATE** |  |
| RCT | Bias due to confounding | PN | Low |  | robust analysis methods to control for potential confounders |
|  | Bias in selection of participants | PY | Moderate |  | authors described uptake of intervention as low, which resulted in a motivated group of participants being included in study (who are likely not representative of target population) |
|  | ***At intervention*** |  |  |  |  |
|  | Bias in classification of interventions | PN | Low |  | intervention groups clearly defined |
|  | ***Post-intervention*** |  |  |  |  |
|  | Bias due to deviations from intended interventions | PY | Moderate |  | patients were tested on a cycle ergometer but not everyone trained on a bicycle & consequently both the baseline and follow-up tests lacked exercise specificity, this could have led to an underestimation of changes in aerobic fitness in those who chose running or swimming as training modality |
|  | Bias due to missing data | PY | Moderate |  | only 79% adherence to intervention |
|  | Bias in measurement of outcomes | PY | Moderate |  | only sports physicians who administered the tests were blinded, the other assessors and patients were not |
|  | Bias in selection of reported results | PN | Low |  | reported results correspond to all intended outcomes, analyses, and subcohorts |
| *Boele (2018)^32^* | ***Pre-Intervention*** |  |  | **MODERATE** |  |
| RCT | Bias due to confounding | PN | Low |  | robust analysis methods to control for potential confounders |
|  | Bias in selection of participants | PN | Low |  | used few exclusion criteria, nationwide recruitment across Netherlands, selection of participants into study was not based on participant characteristics observed after the start of the intervention |
|  | ***At intervention*** |  |  |  |  |
|  | Bias in classification of interventions | PN | Low |  | intervention groups clearly defined |
|  | ***Post-intervention*** |  |  |  |  |
|  | Bias due to deviations from intended interventions | PY | Moderate |  | neither researchers nor participants were blinded to group allocation, unexpected high attrition from study |
|  | Bias due to missing data | PY | Moderate |  | only about 40% of participants adhered to intervention, which may lead to overestimation of some results |
|  | Bias in measurement of outcomes | PY | Moderate |  | some outcomes are patient reported, outcome assessors are aware of intervention status |
|  | Bias in selection of reported results | PN | Low |  | reported results correspond to all intended outcomes, analyses, and subcohorts |
| *Milbury (2019)^28^* | ***Pre-Intervention*** |  |  | **MODERATE** |  |
| RCT | Bias due to confounding | PN | Low |  | robust analysis methods to control for potential confounders |
|  | Bias in selection of participants | PY | Moderate |  | only included high KPS patients, which contributed to selection bias |
|  | ***At intervention*** |  |  |  |  |
|  | Bias in classification of interventions | PN | Low |  | intervention groups clearly defined |
|  | ***Post-intervention*** |  |  |  |  |
|  | Bias due to deviations from intended interventions | PY | Moderate |  | neither assessors nor participants were blinded |
|  | Bias due to missing data | PN | Low |  | 95% of dyads completed T2 assessments, low missing data |
|  | Bias in measurement of outcomes | PY | Moderate |  | outcome assessors aware of intervention status b/c they used PROs |
|  | Bias in selection of reported results | PN | Low |  | reported results correspond to all intended outcomes, analyses, and subcohorts |

*Abbreviations:* Y: yes, PY: probably yes, N: no, PN: probably no
